# Imidazole-based tetrachloridoferrate(iii) magnetic ionic liquids: structural insights, photosensitizing potential, and selective anti-glioblastoma activity

**DOI:** 10.1039/d6ra05375k

**Published:** 2026-08-21

**Authors:** Eugenia Stingaci, Natalia Sucman, Mariana Neamtu, Svetlana G. Baca, Victor Ch. Kravtsov, Jan van Leusen, Paul Kögerler, Leentje Persoons, Dirk Daelemans, Steven De Jonghe, Fliur Z. Macaev

**Affiliations:** a Laboratory of Organic Synthesis, Institute of Chemistry, Moldova State University 3 Academiei str. MD-2028 Chisinau R. Moldova fliur.macaev@sti.usm.md natalia_sucman@yahoo.com; b Department of Exact and Natural Sciences, Institute of Interdisciplinary Research, “Alexandru Ioan Cuza” University Bv. Carol I, no. 11 700506 Iaşi Romania; c Laboratory of Physical Methods of Solid State Investigation “Tadeusz Malinowski”, Institute of Applied Physics, Moldova State University 5 Academiei str. MD-2028 Chisinau R. Moldova; d Institute of Inorganic Chemistry, RWTH Aachen University Landoltweg 1 D-52074 Aachen Germany; e Molecular Genetics and Therapeutics in Virology and Oncology Research Group, Rega Institute for Medical Research, Department of Microbiology, Immunology and Transplantation, KU Leuven Herestraat 49, P.O. Box 1048 B-3000 Leuven Belgium; f Molecular, Structural and Translational Virology Research Group, Rega Institute for Medical Research, Department of Microbiology, Immunology and Transplantation, KU Leuven Herestraat 49, P.O. Box 1049 B-3000 Leuven Belgium

## Abstract

Tetrachloridoferrate-based ionic liquids represent an emerging class of functional materials with promising magnetic and biological properties, yet their potential as photosensitizers and anticancer agents remains underexplored. We report the synthesis of new imidazole-based tetrachloridoferrate(iii) salts and a comprehensive evaluation of their structural, magnetic, photochemical, and cytotoxic properties. The target ionic liquids were prepared through the quaternization of imidazole derivatives, followed by anion exchange with FeCl_3_. Structural analysis *via* single-crystal X-ray diffraction of selected compounds revealed distinct non-covalent interaction patterns and charge distribution governing stability, while magnetic measurements demonstrated weak antiferromagnetic exchange interactions along the tetrachloridoferrate chains. Furthermore, ESP experiments revealed efficient photosensitized singlet oxygen generation under UV irradiation, which correlates with photodegradation of bisphenol A. Biological evaluation across a broad panel of solid and haematological cancer cell lines identified remarkable, selective cytotoxicity toward the LN229 glioblastoma cell line, with IC_50_ values as low as 0.70–1.50 µM, while no significant activity was observed against other glioblastoma models or normal PBMCs. These findings highlight imidazolium tetrachloridoferrates as compelling candidates for further investigation in oxidative-stress-based anticancer strategies.

## Abstract 2

The tetrachloridoferrate salts of 1*H*-imidazole derivatives displayed selective cytotoxicity against the LN229 glioblastoma cell line with IC_50_ values in the low micromolar range (1.0–4.0 µM), which is comparable to the reference drug etoposide.

## Graphical abstract

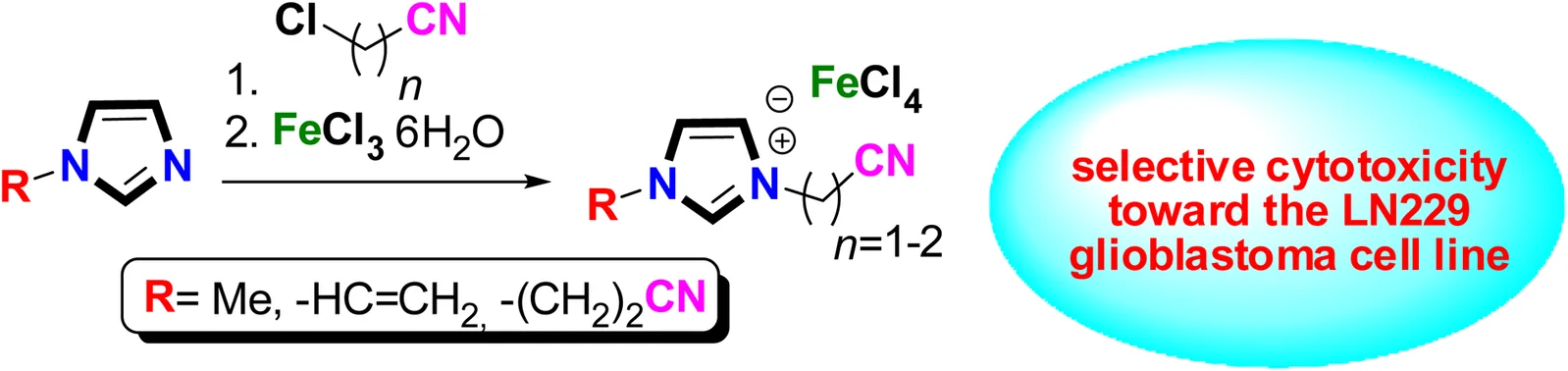

## Introduction

Ionic liquids (ILs) constitute a dynamic and rapidly evolving field within modern organic chemistry, owing to their remarkable physicochemical properties and diverse range of applications.^1,2^ Although ILs as a class are extensively documented in the literature, those incorporating a tetrachloridoferrate anion remain relatively rare. They are generally described as magnetic materials,^3–5^ catalysts for organic synthesis,^6,7^ or surfactant-like systems applicable in various technological processes, including extraction and recovery of valuable compounds.^3–9^ Beyond their traditional roles, an increasing number of ionic liquids have been recognized as biologically active entities rather than inert solvents, exhibiting antimicrobial, antifungal, and anticancer activities.^10^ In particular, imidazolium-based ILs have demonstrated pronounced cytotoxic effects against various cancer cell lines, which have been associated with membrane disruption, mitochondrial dysfunction, and the induction of oxidative stress.^11–16^ These observations have stimulated growing interest in the rational design of functional ionic liquids as potential anticancer agents.

Singlet oxygen (^1^O_2_), derived from triplet oxygen (^3^O_2_), is a unique reactive oxygen species widely used in the synthesis of chemicals and biologically active compounds,^17–21^ as well as in photodynamic therapy (PDT).^22,23^ Generally, ^1^O_2_ is generated *via* photosensitization under light irradiation, a process that has attracted considerable attention^24–29^ across diverse applications.^30^ As a non-invasive treatment modality, PDT is utilized for various oncological and non-neoplastic conditions, including psoriasis, autoimmune and inflammatory skin disorders, and age-related macular degeneration, particularly for patients ineligible for conventional therapies. This approach relies on the systematic or topical administration of a photosensitizer (PS) that accumulates in target cells and is subsequently activated by a laser light beam of a specific wavelength matching the absorption band of the PS.^31,32^ Consequently, effective PDT outcomes require three essential components: PS, endogenous molecular oxygen (^3^O_2_), and visible light.^33–35^

Briefly, light absorption excites the PS to a triplet excited state, enabling energy transfer to ^3^O_2_ and generating cytotoxic ^1^O_2_*via* a type II photochemical reaction.^35^ The ^1^O_2_ generated *in situ* induces damage to the plasma membrane or DNA of malignant cells, ultimately leading to cell death.^31^ Because this mechanism depends on a photosensitizer, numerous light-absorbing molecules have been evaluated, including transition metal-based complexes,^36–38^ metal-free dyes,^22^ and tetrapyrrole-based compounds such as porphyrins,^39–41^ chlorins,^42,43^ bacteriochlorins,^44^ and phthalocyanines.^31,45^

Alternatively, the generation of ^1^O_2_ by tetrachloridoferrate(iii) presents a distinct pathway that can significantly affect biological activity, including the cytotoxic properties against various tumor cell types without a strict reliance on external light activation.^17,18^ However, the efficacy of this iron-based approach heavily depends on the antioxidant potential of the cells, the characteristics of iron metabolism, the level of hypoxia, and membrane permeability.^46,47^ In addition to their biological activity, tetrachloridoferrate(iii) compounds possess intrinsic magnetic properties. This multifunctionality offers significant potential for a theranostic approach, combining targeted drug delivery *via* external magnetic fields and contrast enhancement in magnetic resonance imaging (MRI) with localized therapeutic action.

Driven by these considerations, the aim of this study was to synthesize novel imidazole-based tetrachloridoferrate(iii) salts and to investigate their key properties, including magnetic behaviour, singlet-oxygen generation, and cytotoxic activity.

## Results and discussion

### Synthesis

**Scheme 1:**
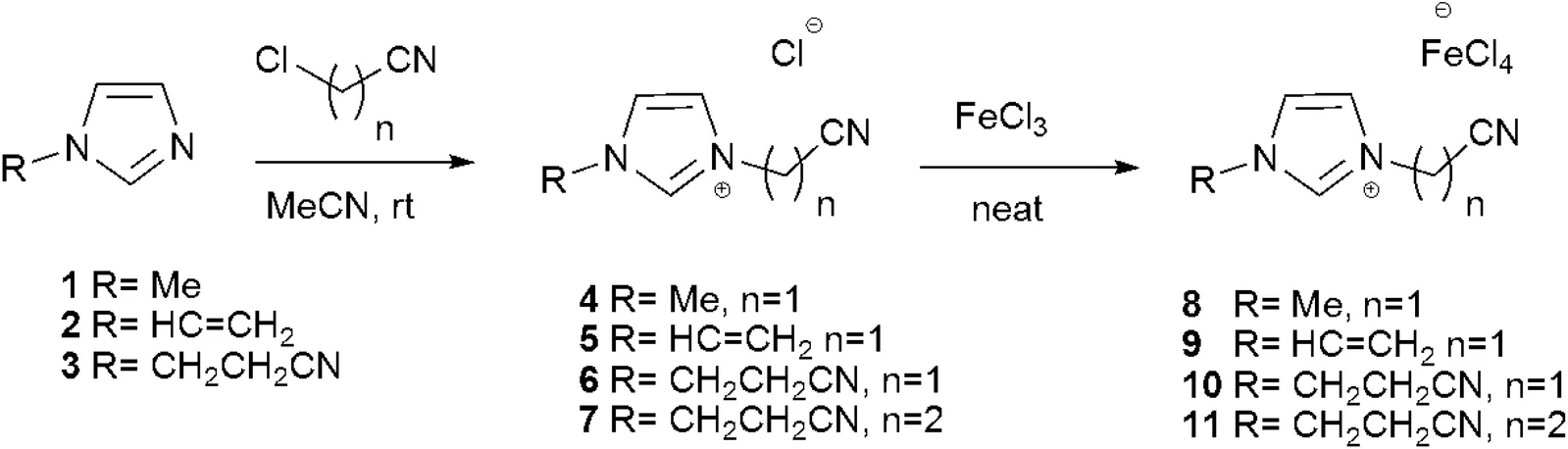
Synthesis of imidazole-based tetrachloridoferrate(iii) 8–11.

In continuation of our previous investigations into cytotoxic ionic liquids,^14,48,49^ we have focused on new imidazolium tetrachloridoferrates as promising agents for comprehensive screening against both solid tumor and hematological cancer cell lines. The synthesis of these target compounds was carried out according to the protocol outlined in Scheme 1.

Commercially available 1-methylimidazole (1) and 1-vinylimidazole (2) were employed as starting materials for the synthesis of the target ionic liquids. The preparation of compound 3, which also served as a precursor for subsequent quaternization reactions, involved the nucleophilic addition of imidazole to acrylonitrile in toluene under gentle heating, catalyzed by triethylamine.

In the next stage, intermediate ionic liquids were obtained *via* a classical quaternization reaction of imidazoles with an activated alkyl halide. Using this approach, imidazolium salts 4, 5, and 6 were synthesized employing chloroacetonitrile as the alkylating agent.

The synthesis of compound 7, however, proved unexpectedly challenging. Multiple attempts yielded inconsistent results, with significant variations in both yield and purity. Analytical examination of the reaction mixtures revealed that, alongside the targeted ionic liquid, the side product imidazolium propionitrile hydrochloride, was consistently formed. In certain cases, trace amounts of imidazolium hydrochloride were also detected. While the presence of the latter could be attributed to trace amounts of unreacted starting imidazole carried over from the previous reaction, the formation of imidazolium propionitrile hydrochloride indicates that decomposition pathways of compound 7 are active under the reaction conditions. These pathways appear to involve the elimination of acrylonitrile and hydrogen chloride, leading to the formation of the undesired salt. Alternatively, the formation of hydrogen chloride may arise from the decomposition of 3-chloropropionitrile. At this stage, both mechanistic scenarios remained plausible yet difficult to definitively substantiate. Through systematic variation of reaction time, molar ratios, solvents, and purification methods, we identified optimized conditions that enabled the isolation of the desired product 7 with satisfactory purity and reproducibility. The identity and purity of all synthesized precursors 4–7 were confirmed by ^1^H, ^13^C, and DEPT NMR (Fig. S1–S11) and FT-IR (Fig. S12–S15) spectroscopy, as well as by elemental analysis.

The subsequent step consisted of replacing the chloride counterion with tetrachloridoferrate(iii). For this purpose, a mixture of the corresponding imidazolium chloride and FeCl_3_ was left undisturbed at room temperature for two days. The resulting products were dried under reduced pressure to remove residual water, and analytical samples of salts 8–11 were obtained *via* recrystallization from an appropriate solvent.

### Crystal structures of 9 and 10

**Fig. 1:**
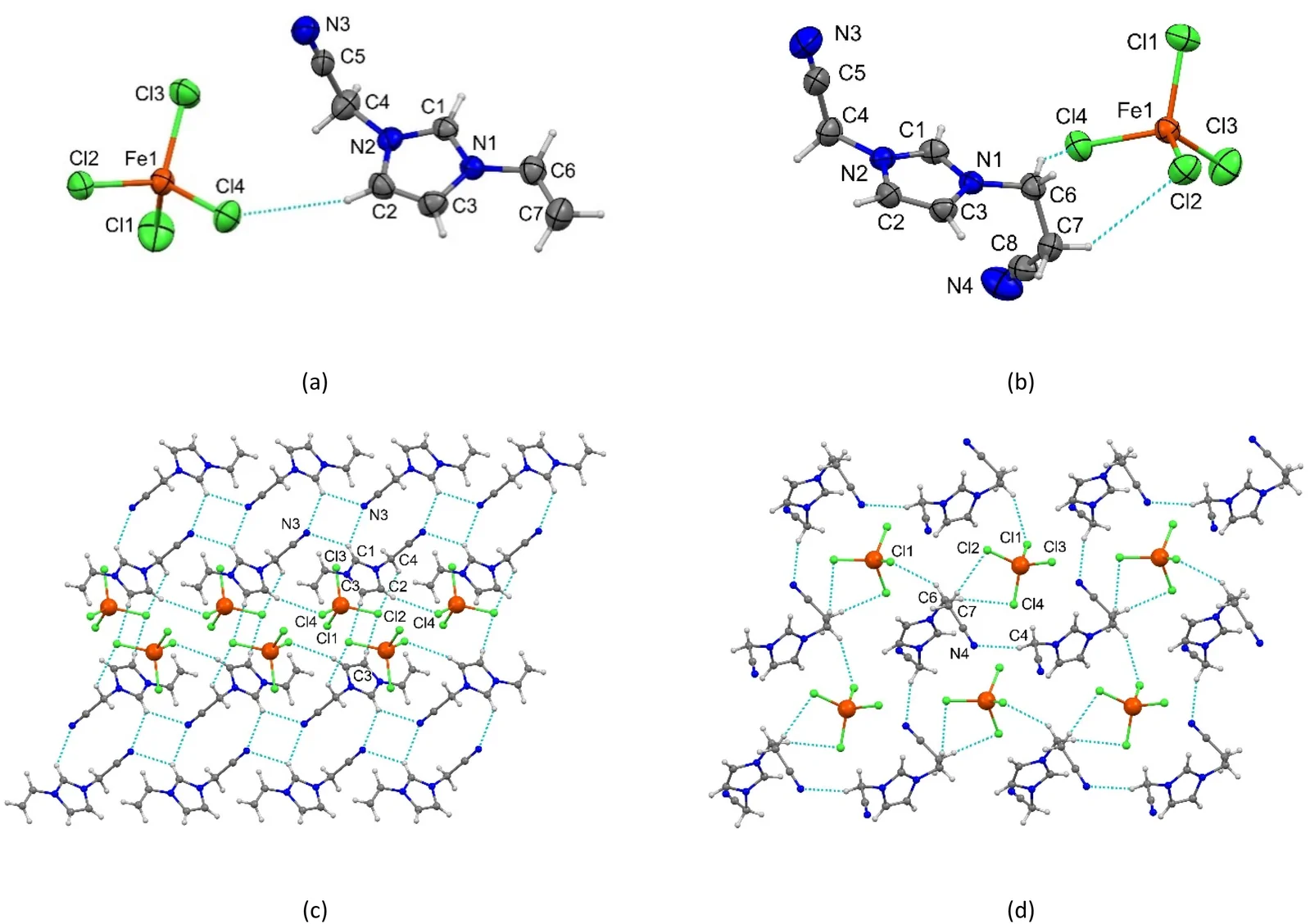
Molecular structure of the asymmetric units of 9 (a) and 10 (b) with numbering scheme, thermal ellipsoids are drawn at 50% probability level. Fragments of crystal packing illustrate the formation of supramolecular layers in 9 (c) and 10 (d).

Single-crystal X-ray analysis reveals that compound 9 crystalizes in the *P*1̄ space group of the triclinic system with the unit cell parameters *a* = 6.6339(6), *b* = 9.2678(9), *c* = 11.7121(10) Å, *α* = 104.408(8), *β* = 92.424(7), *γ* = 1 07.627(8)°, *V* = 659.26(11) Å^3^, whereas compound 10 crystalizes in the *P*2_1_/*c* group of the monoclinic system with *a* = 9.8765(5), *b* = 12.2383(4), *c* = 12.5215(6) Å^3^, *β* = 108.106(5)°, *V* = 1438.55(12) Å^3^ (Table S1). In the crystal structure of both compounds, the asymmetric unit cell contains the ion pair of one imidazolium-based cation, [C_7_H_8_N_3_]^+^ (9) or [C_8_H_9_N_3_]^+^ (10), and one tetrachloridoferrate(iii) anion, [FeCl_4_]^−^. The molecular structures of 9 and 10 are shown in Fig. 1a and b. The linear cyanomethyl group in 9 is pointed approximately perpendicular to the plane of the imidazole fragment and torsion angle C1–N2–C4–C5 equals 95.5(4)°, while the ethenyl branch is close to this plane and the torsion angle C1–N1–C6–C7 equals 165.8(4)°. The cyanomethyl group in 10 adopts the similar orientation and torsion angle C1–N2–C4–C5 equals 97.8(3)°. The cyanoethyl group is oriented opposite the cyanomethyl group in relation to the imidazole plane. This differs from the orientation of this branch in the same cation in the bromine analog structure.^50^ The torsion angles C1–N1–C6–C7 and N1–C6–C7–C8 equal 119.2(3)° and −64.3(3)°, respectively.

In the crystal, the arrangement of the ionic components is supported by weak C–H⋯N cation⋯cation and C–H⋯Cl charge-assisted cation⋯anion interactions, Table S3. In the structure of 9, all of the carbon atoms of the imidazole moiety are involved in these interactions: C1 is involved in bifurcated cation⋯cation C–H⋯N interactions and C2 and C3 are involved in cation⋯anion C–H⋯Cl interactions. In contrast, none of the carbon atoms of the imidazole moiety participate in such interactions in the crystal structure of 10. In both structures, all methylene carbon atoms participate in these interactions. These interactions unite the ionic components in the supramolecular layer parallel to the (0 −1 1) crystallographic plane in 9 and the (0 0 1) plane in 10, respectively, Fig. 1c and d. The layers are stacked in a parallel manner, with no interatomic distances between the layers shorter than the sum of the corresponding van der Waals radii.

Single-crystal X-ray analysis provided deeper insight into the structural features of the obtained salts. In compound 9, all carbon atoms of the imidazole ring are involved in non-covalent interactions with the anion. In contrast, in compound 10, none of the imidazole carbon atoms participate in such interactions; instead, both methylene carbons of the propionitrile fragment show clear involvement. This observation indicates that the positive charge of the cation is localized neither on the imidazole ring nor on the carbon adjacent to the chloroacetonitrile moiety, but is rather shifted toward the C-2 and C-3 positions of the propionitrile chain.

Consequently, the distribution of positive charge is displaced along the carbon chain. This phenomenon allows extrapolation to the case of the symmetric ionic liquid precursor 7. The localization of charge on the carbon chain rationalizes the observed elimination of hydrogen chloride, which leads to the formation of the corresponding secondary imidazolium salt. Thus, we propose that the formation of this side product is not a competing reaction, but instead a sequential step involving decomposition of the primary product.

This mechanistic hypothesis retroactively justifies the optimization strategy for the synthesis of 7, where lowering the reaction temperature to room temperature and avoiding prolonged stirring suppressed these decomposition pathways. These precise modifications enabled the isolation of the desired chloride 7 in pure form, which was then successfully employed in the subsequent synthesis of tetrachloridoferrate(iii) salt 11.

### Spectroscopic and spectrometric characterization

The FT-IR spectra (Fig. S16–S19) of the obtained compounds 8–11 display characteristic absorption bands, including a broad stretching region between 3200 cm^−1^ and 2800 cm^−1^ corresponding to C–H stretching vibrations. The pronounced broad signal at 3387 cm^−1^ suggests the presence of crystallization water in the crystal structure. Two medium-intensity bands at 3094 cm^−1^ and 3061 cm^−1^ belong to the stretching vibrations of the C–H bonds of the vinyl group in the case of compound 9 (Fig. S17), whereas the bands at 2980 cm^−1^ and 2919 cm^−1^ correspond to aliphatic stretching vibrations. The band at 1653 cm^−1^ is associated with the stretching vibrations of the C=C bond of the vinyl group. Moreover, the band at 1670 cm^−1^ corresponds to imidazole C=C stretching vibrations, while a weak band at 2257 cm^−1^ and a band at 1177 cm^−1^ correspond to the stretching vibration frequency of the CN group and the aliphatic C–N bond, respectively. Additionally, two strong signals at 930 cm^−1^ and 958 cm^−1^ and two medium signals at 1410 cm^−1^ and 1287 cm^−1^ belong to the out-of-plane C–H bending vibrations of the vinyl group. The only significant difference between the FT-IR spectra of tetrachloridoferrates and those of the initial chlorides in the 4000–800 cm^−1^ region is the shift of the C–H bond signals to higher wavenumbers (Fig. S12–S15).

Attempts to obtain single crystals of tetrachloridoferrates 8 and 11 suitable for X-ray diffraction analysis were unsuccessful. In addition, NMR spectroscopy could not be used due to the paramagnetic nature of these species. Therefore, their structures were confirmed by high-resolution mass spectrometry techniques, specifically ESI(±) and ESI Q/TOF MS (Fig. S20–S29), as well as by elemental analysis.

In the ESI(+) mode, characteristic peaks corresponding to the imidazolium cations were detected with 100% intensity at *m*/*z* 122 ([C_6_H_8_N_3_]^+^ for 8), 134 ([C_7_H_8_N_3_]^+^ for 9), 161 ([C_8_H_9_N_4_]^+^ for 10), and 175 ([C_9_H_11_N_4_]^+^ for 11). In the case of ESI(+) Q/TOF MS, the formation of doubly charged protonated imidazolium cations ([M + 2H]^2+^) was additionally observed (Fig. S20, S23, S26 and S29). Furthermore, the ESI(+)-Q/TOF mass spectrum of compound 10 contains a group of signals with a maximum at *m*/*z* 357, whose isotopic distribution is consistent with an aggregate composed of two imidazolium cations and one chloride ion (Fig. S27). Similarly, the signals observed at *m*/*z* 553 were assigned to a trimeric species comprising three imidazolium cations and two chloride anions.

Concurrently, the ESI(−) mode confirmed the presence of the tetrachloridoferrate counteranion in all cases. In the ESI(−) mass spectra, a prominent signal for the [FeCl_4_]^−^ anion was consistently observed at *m*/*z* 197, with an isotopic distribution pattern that perfectly matches the theoretical simulation. Additionally, the ESI(−) mass spectra revealed the formation of heavy dimeric aggregates; specifically, peaks observed at *m*/*z* 529 for compound 9, assigned to the [(C_7_H_8_N_3_)(FeCl_4_)_2_]^−^ species, and at *m*/*z* 556 for compound 10, attributed to the [(C_8_H_9_N_4_)(FeCl_4_)_2_]^−^ species.

### Magnetic properties of 9 and 10

**Fig. 2:**
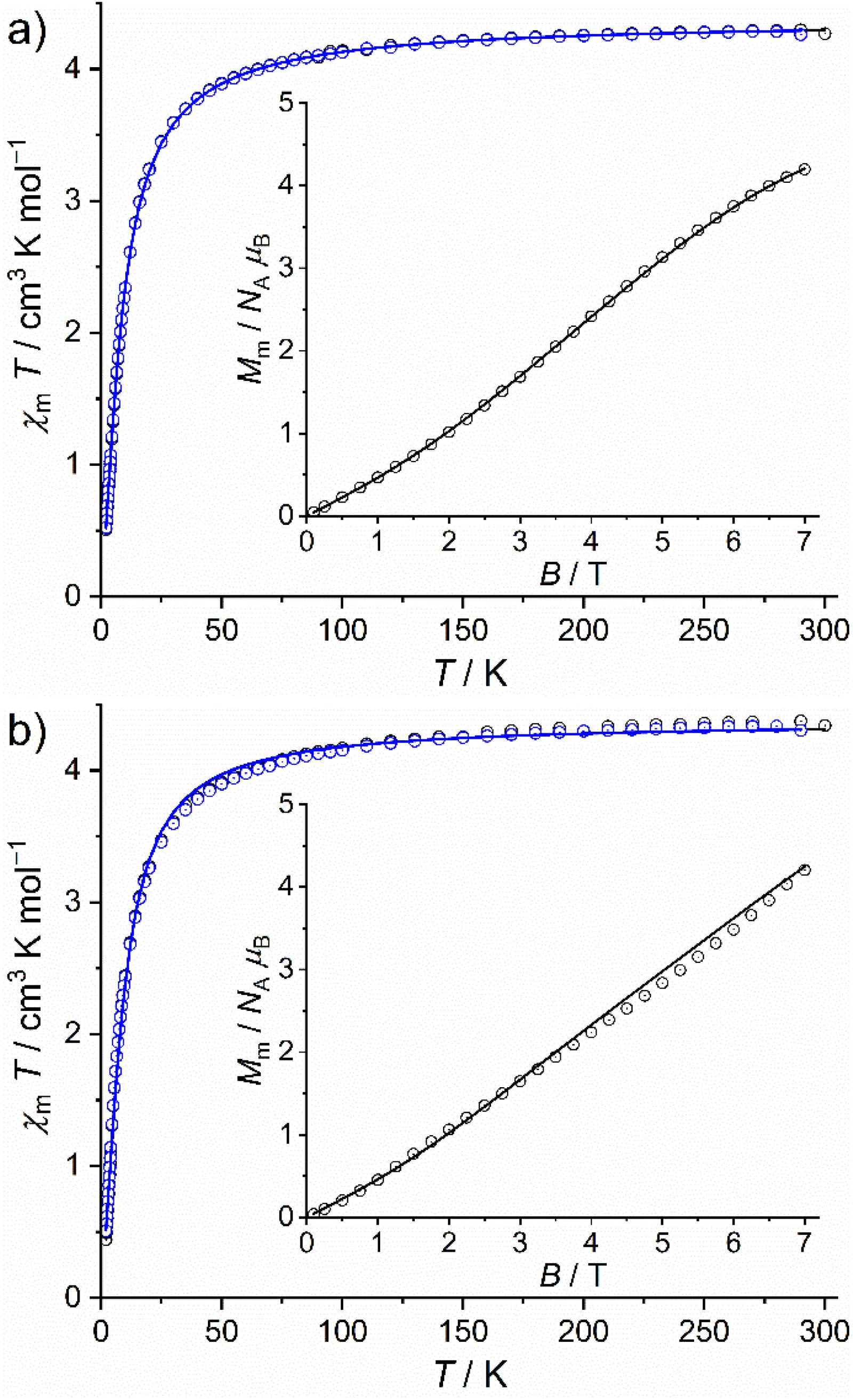
Magnetic measurements of 9 (a) and 10 (b): *χ*_m_*T vs. T* at 0.1 T (black) and 1.0 T (blue) and molar magnetization *M*_m_*vs. B* at 2.0 K (insets). Experimental data are represented by open circles, least squares fits by solid lines.

SQUID magnetometry data are presented in Fig. 2 as plots of *χ*_m_*T versus T* under applied magnetic fields of 0.1 and 1.0 T, along with molar magnetization plots *M*_m_*versus B* at 2.0 K. At 300 K, the *χ*_m_*T* values at 0.1 T are 4.30 (9) and 4.34 cm^3^ K mol^−1^ (10), respectively. Both values are within the expected range of 4.06–4.50 cm^3^ K mol^−1^ for an isolated high spin Fe^3+^ center,^51^ yet slightly below the theoretical value of 4.38 cm^3^ K mol^−1^ of a free spin-only center with total spin *S* = 5/2 and *g* = *g*_e_ ≈ 2.0. Upon cooling the compounds, *χ*_m_*T* continuously decreases featuring a drop off at *T* < 50 K. At 2.0 K, *χ*_m_*T* values reach 0.51 (9) and 0.44 cm^3^ K mol^−1^ (10), respectively. The corresponding data at 1.0 T are very similar to the 0.1 T data set showing 0.52 (9) and 0.51 cm^3^ K mol^−1^ (10) at 2.0 K. Magnetization curves recorded at 2.0 K display an almost linear increase from the origin to 4.2 *N*_A_*µ*_B_ at 7.0 T for both compounds, remaining distinctly below the theoretical saturation value of 5.0 *N*_A_*µ*_B_. Closer inspection of these magnetization curves reveals weakly sigmoidal features, particularly in the case of 9. The observed data may come at a surprise since the [FeCl_4_]^−^ moieties are somewhat isolated according to structural data (and the possibility of a tetrahedral 3d^5^ spin crossover complex made of Fe^3+^ and four chlorido ligands can be safely excluded).

For isolated [FeCl_4_]^−^ complexes, the *χ*_m_*T vs. T* data are constant above 10 K, and show slight decreasing values of *χ*_m_*T* below that temperature due to the Zeeman effect. Additionally, the magnetization curve is well saturated at magnetic fields larger than about 3 T. Therefore, the data of 9 and 10 indicate the presence of antiferromagnetic exchange interactions. Note that such behavior is not unprecedented for frozen ionic liquids containing [FeCl_4_]^−^ moieties, see *e.g.* ref. 52 reporting similar observations. Although structural data indicate no bridging ligands between two Fe^3+^ centers (and no direct Fe–Fe bonding), certain [FeCl_4_]^−^ moieties form chains made of somewhat close Fe^3+^ ions, and thus very weak exchange interactions may arise. They may be even enhanced, since some Cl^−^ ligands of two [FeCl_4_]^−^ moieties are in close proximity of about 3.8 Å. In (9), these chains show alternating distances of 5.896 Å and 7.865 Å between two Fe^3+^ ions, while in 10 these distances are 5.635 Å and 7.704 Å.

To shed some light on the nature of the exchange interactions, we fit the data employing the spin-only option of the computational framework CONDON.^53^ The least-squares fits to an effective isotropic spin Hamiltonian (using the “−2*J*” notation, and *g*_eff_ = 2.0) yield the data represented as solid lines in Fig. 2. Note that the results should be understood as rough estimations, since we assume the interactions to extend along the aforementioned chains of identical *S* = 5/2 centers without further proof. Due to the alternating distances between two Fe^3+^ centers, we allow for two different exchange interaction parameters *J*_1_ and *J*_2_. To check for relevant interactions with centers not part of the same chain, we additionally allow for a further fit parameter describing these interactions in a mean-field approach (*zJ*_mf_, where *z* is the number of neighboring centers not part of the chain). The found values for the Heisenberg parameters are *J*_1_ = (−0.58 ± 0.02) cm^−1^, *J*_2_ = (−0.14 ± 0.01) cm^−1^ and *zJ*_mf_ = (−0.01 ± 0.01) cm^−1^ (for 9, with a relative root mean square error SQ = 0.44%). For 10, we found *J*_1_ = (−0.67 ± 0.02) cm^−1^, *J*_2_ = (−0.32 ± 0.02) cm^−1^ and *zJ*_mf_ = (+0.08 ± 0.06) cm^−1^ (SQ = 4.1%). We note that we have also considered alternative coupling scenarios such as a single-*J* chain (*J*_1_ = *J*_2_) or isolated dimers, which however resulted in worse fits to the experimental *χ*_m_*T vs. T* and *M*_m_*vs. B* data. Therefore, we find very weak antiferromagnetic exchange interactions within a chain. They are in agreement with alternating distances, since shorter distances translate to stronger interactions, in particular, comparing the determined parameters of both compounds. Further interactions with centers not part of the chain are very small, or even non-existent within the error margins of the mean-field parameter. At this point, we cannot infer from data whether there is long-range magnetic ordering, as *e.g.* suggested in ref. 52, or not. However, considering the small magnitudes of the exchange parameters and structural data, we rather exclude this possibility, although neutron diffraction measurements could ultimately answer this question.

### ESR tests

**Fig. 3:**
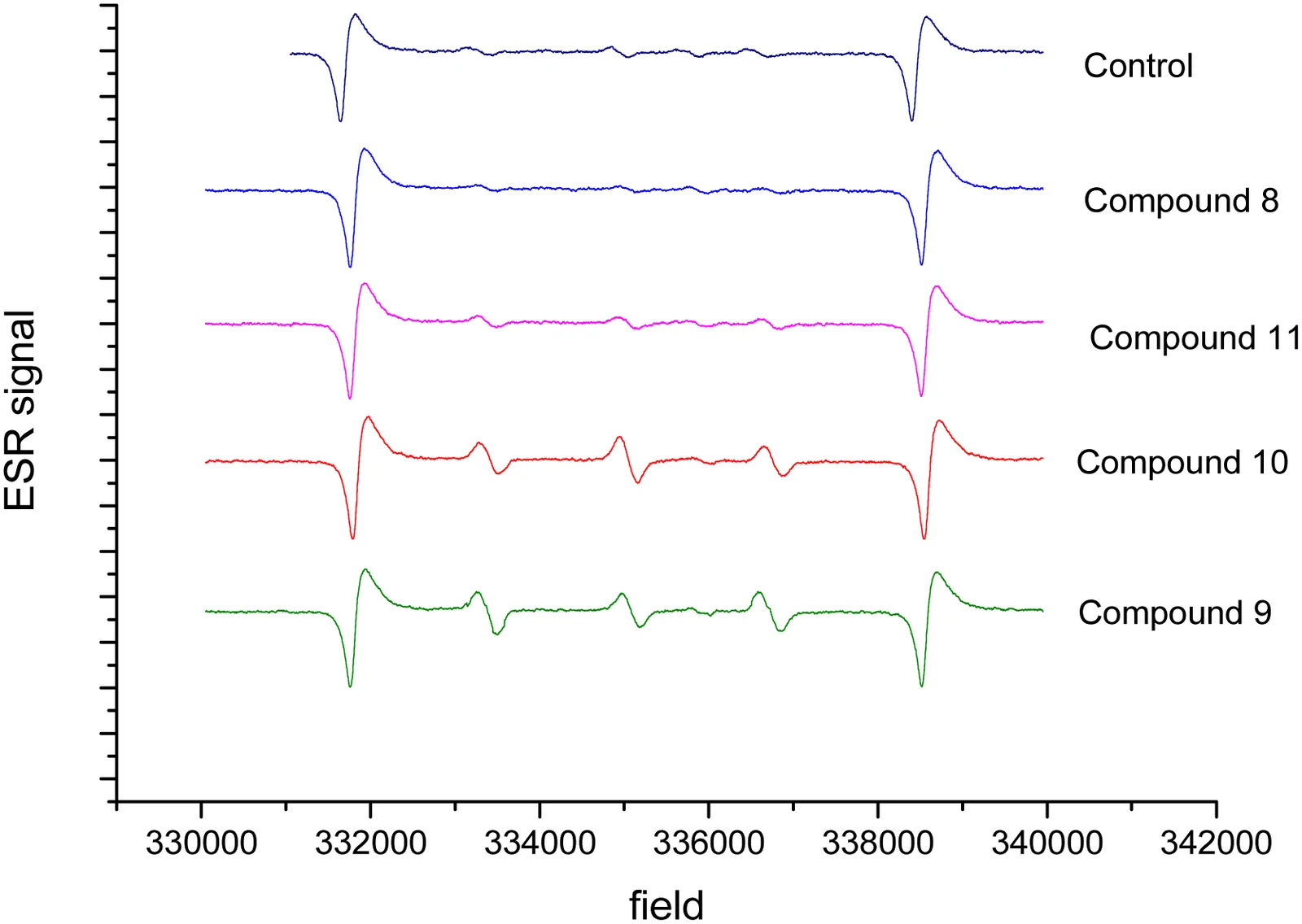
ESR spectra acquired after 60 min of UV illumination (*λ* = 365 nm) of a TMP–OH solution in the presence of the compounds and of a control solution.

The property as a photosensitizer of the studied compounds to generate singlet oxygen was also investigated. The formation and quenching of the photosensitized singlet molecular oxygen, ^1^O_2_, generated by the compounds, was investigated by ESR using 2,2,6,6-tetramethyl-4-piperidinol (TMP–OH) as spin trap. The ESR spectra obtained from the samples containing TMP–OH in the presence of compounds and in its absence consist of three equidistant and equi-intense hyperfine lines (Fig. 3).

After a 60 min exposure to UV light, the solution containing the compounds as well as the control solution (containing only TMP–OH), showed a characteristic ESR signal (Fig. 3). The compounds 9 and 10 have been shown the highest signal, with a quantum yield of 0.0118 and 0.0088, respectively. The signal intensity of the 4-hydroxy-2,2,6,6-tetramethylpiperidine-1-oxyl (TEMPOL) spectra acquired for the compounds is larger than for the control solution. This suggests that, under exposure to UV light, the compounds contributed to the overall singlet oxygen formation. The ESR experiments point to the formation of ^1^O_2_ by the studied compounds in aqueous suspension. Singlet molecular oxygen (O_2_(^1^Δ_g_), denoted throughout as ^1^O_2_), the lowest electronic excited state of molecular oxygen, is an important oxidizing species in chemical processes and one of the main activated species responsible for the damaging effects of light on biological systems (photodynamic effects).^54^ This activated metastable state has a different and unique reactivity than the ground triplet state (O_2_(^3^Σ_g_), denoted throughout as ^3^O_2_) and has been attracting interest in both practical and fundamental aspects. Photosensitization is primarily responsible for the production of ^1^O_2_.^55^ This process can be summarized as follows: a sensitizer is promoted by the absorption of a photon to an electronically excited singlet state and can subsequently undergo intersystem crossing to generate a longer lived excited triplet state. Singlet oxygen may be then produced by energy transfer to dissolved molecular oxygen.^28,29,55,56^ The property of compounds to generate singlet oxygen species has been also confirmed in photodegradation of bisphenol A. More than 80% of bisphenol A have been reduced in the presence of compounds 9 and 10. Our results are in agreement with those reported by different authors.^14,57,58^

### Cytotoxicity

The newly synthesized imidazole-based tetrachloridoferrate salts 8–11 were evaluated for their anticancer potential across a broad panel of solid tumour and haematological cancer cell lines (Table 1). Etoposide (a DNA topoisomerase II inhibitor) was included as positive control, yielding potent activity across all eight tumour cell lines, consistent with literature reports. In contrast, compounds 8–10 completely lacked cytotoxic activity against all other solid tumour cell lines (Capan-1, HCT-116, and NCI-H460) and haematological malignancies (HL-60, K562, and Z138). To determine if compounds 8–11 exhibited selective cytotoxicity *versus* cancer cells, they were also evaluated for potential cytotoxicity against normal (*i.e.* non-cancerous) peripheral blood mononuclear cells (PBMCs) (Table 2). Whereas the reference compound showed pronounced cytotoxicity in PBMCs, compounds 8–11 show only minimal cytotoxicity against PBMCs (IC_50_ values ranging from 10.3 to 59.8 µM), with compound 8 emerging as the most selective candidate.

**Table 1:** Cytotoxicity of the compounds (IC_50_, µM; data presented as mean ± S.D.)^a^

Compound	Solid tumour cell lines				Haematological cell lines			
	Capan-1	HCT-116	LN229	NCI-H460	DND-41	HL-60	K562	Z138
8	>100	>100	1.35 ± 0.55	>100	>100	>100	>100	>100
9	>100	>100	0.70 ± 0.10	>100	>100	>100	>100	>100
10	>100	>100	0.75 ± 0.15	>100	>100	>100	>100	>100
11	>100	>100	1.50 ± 0.60	>100	>100	>100	>100	>100
Etoposide	0.08 ± 0.04	1.5 ± 0.2	2.05 ± 0.95	6.2 ± 1.6	0.02 ± 0.01	0.3 ± 0.1	2.15 ± 0.45	0.35 ± 0.05

a Capan-1 – pancreatic adenocarcinoma; HCT-116 – colorectal carcinoma; LN229 – glioblastoma; NCI-H460 – lung carcinoma; DND-41 – acute lymphoblastic leukemia; HL-60 – acute myeloid leukemia; K562 – chronic myeloid leukemia; Z138 – non-Hodgkin lymphoma.

**Table 2:** Cytotoxicity of the compounds (IC_50_, µM; data presented as mean ± S.D.)^a^

Compound	Cytotoxicity		
	PBMC	U138 MG	U87 MG
8	59.8 ± 2.00	>100	>100
9	19.8 ± 1.80	>100	>100
10	10.3 ± 0.95	>100	>100
11	18.9 ± 1.60	>100	>100
Etoposide	3.0 ± 0.50	7.95 ± 0.25	1.9 ± 0.0

a PBMC – peripheral blood mononuclear cells; LN229, U138 MG, and U87 MG – human glioblastoma cell lines with distinct genetic profiles and morphologies.

Because of their selective antitumoral activity against the LN229 glioblastoma cell line, compounds 8–11 were also evaluated against other glioblastoma cell lines, including the U138 MG and U87 MG cell lines. Parallel assays reconfirmed cytotoxicity against LN229 cells (Table 2).

Remarkably, compounds 8–11 exhibited selective cytotoxicity toward the LN229 glioblastoma cell line, whereas no significant activity was observed against the U87 MG and U138 MG cell lines under the tested conditions. This finding highlights the differential sensitivity of glioblastoma models to the synthesized tetrachloridoferrate(iii) ionic liquids. Since glioblastoma cell lines differ substantially in their genetic background, molecular characteristics, and therapeutic response, the observed selectivity is likely associated with intrinsic biological differences among these models.^59^ However, the molecular basis of this selective activity cannot be established from the present data. Further studies will be required to elucidate the mechanism underlying the differential response of LN229, U87 MG, and U138 MG cells.

## Experimental section

### Materials and methods

Chemicals were of reagent grade and used as received. Removal of all solvents was carried out under reduced pressure. ^1^H, ^13^C NMR, and DEPT spectra (Fig. S1–S11) were recorded for 2% solutions of compounds in DMSO-*d*_6_ on a Bruker-Avance III (400.13 and 100.61 MHz) (Karlsruhe, Germany) spectrometer. Chemical shifts (*δ*) are given in parts per million (ppm), referring to the signal center of the deuterated solvent peaks as reference. The signal observed at *δ* 3.4 ppm in the ^1^H NMR spectra (Fig. S1, S4 and S7) corresponds to residual water in the deuterated solvent rather than to moisture present in the synthesized ionic liquids. IR spectra were recorded on a Spectrum 100 FT-IR spectrophotometer (PerkinElmer, Waltham, MA, USA) using the universal ATR sampling accessory. Elemental analysis (C, H, N) was performed on an Elementar Vario EL analyzer (Santa Clara, CA, USA). Melting points (uncorrected) were determined on a Boetius apparatus (Dresden, Germany). High-resolution electrospray ionization mass spectrometry measurements were performed using two different instruments. For molecular mass determination, the samples were solubilized in acetonitrile and analyzed on an Agilent 6500 Series Accurate-Mass Quadrupole Time-of-Flight (Q-TOF) LC/MS instrument at a flow rate of 0.03 mL min^−1^. The ESI-Q/TOF MS conditions were set as follows: electrospray ionization (positive and negative mode), drying gas flow (N_2_) of 8.0 L min^−1^, drying gas temperature of 325 °C, nebulization pressure of 30 psig, capillary voltage of 4000 V, and fragmentation voltage of 175 V. The mass spectra were recorded in the *m*/*z* range 100–1500. Data were collected and processed using MassHunter Workstation Software Data Acquisition for 6200/6500 Series, version B.07.00 (Agilent Technologies, Inc., Santa Clara, CA, USA). Alternatively, ESI-MS analysis of the samples was performed in both positive and negative ion modes using a ThermoFisher Scientific LTQ-Orbitrap XL mass spectrometer. For these measurements, the samples were prepared by dissolving the compounds in MeOH containing 0.1% acetic acid.

### Typical procedure for the synthesis of imidazolium chlorides 4–7

To a solution of the *N*-substituted imidazole (0.01 mol) in MeCN (10 mL), 2-chloroacetonitrile or 3-chloropropanenitrile (0.01 mol) was added dropwise. The reaction mixture was stirred for 24 hours. The solvent was removed under reduced pressure to give target imidazolium chlorides 4–7.

#### 3-(Cyanomethyl)-1-methyl-1*H*-imidazol-3-ium chloride 4

M.p. = 185–186 °C. IR (*ν*, cm^−1^): 3387, 3177, 3145, 3033, 2979, 2910, 2839, 2421, 2261, 1825, 1769, 1686, 1661, 1622, 1576, 1566, 1442, 1386, 1338, 1255, 1243, 1169, 1106, 1095, 1027, 917, 885, 772, 760. ^1^H NMR (400 MHz, DMSO-*d*_6_) *δ* 9.54 (s, 1H, CH-Im), 7.99 (t, *J* = 1.7 Hz, 1H, CH-Im), 7.87 (t, *J* = 1.6 Hz, 1H, CH-Im), 5.81 (s, 2H, C*H*_2_), 3.91 (s, 3H, Me). ^13^C NMR (101 MHz, DMSO) *δ* 138.2 (Im), 124.8 (Im), 123.0 (Im), 115.3 (CN), 37.1 (CH_2_), 36.6 (Me). Anal. calcd for C_6_H_8_ClN_3_: C, 45.73; H, 5.12; Cl, 22.50; N, 26.66%. Found: C, 45.71; H, 4.98; N, 26.78%.

#### 3-(Cyanomethyl)-1-vinyl-1*H*-imidazol-3-ium chloride 5

M.p. = 164 °C. IR (*ν*, cm^−1^): 3094, 3061, 3034, 2980, 2919, 2257, 1862, 1742, 1670, 1653, 1567, 1555, 1410, 1371, 1355, 1321, 1287, 1177, 958, 930, 870, 764, 745, 717, 661. ^1^H NMR (400 MHz, DMSO-*d*_6_) *δ* 9.91–9.86 (m, 1H, CH-Im), 8.40 (s, 1H, CH-Im), 8.12 (s, 1H, CH-Im), 7.43 (dd, *J* = 15.6, 8.7 Hz, 1H, C*H*-vinyl), 6.06 (dd, *J* = 15.6, 2.4 Hz, 1H, C*H*H-vinyl), 5.79–5.77 (m, 2H, C*H*_2_CN), 5.47 (dd, *J* = 8.7, 2.4 Hz, 1H, CH*H*-vinyl). ^13^C NMR (101 MHz, DMSO) *δ* 137.2 (Im), 129.2 (Im), 123.9 (Im), 120.1 (*C*H=CH_2_), 114.8 (CN), 110.2 (CH=*C*H_2_), 37.6 (*C*H_2_CN). Anal. calcd for C_7_H_8_ClN_3_: C, 49.57; H, 4.75; Cl, 20.90; N, 24.77%. Found: C, 49.74; H, 4.61; N, 24.84%.

#### 1-(2-Cyanoethyl)-3-(cyanomethyl)-1*H*-imidazol-3-ium chloride 6

M.p. = 124–125 °C. IR (*ν*, cm^−1^): 3327, 3276, 3166, 3140, 3126, 3067, 3036, 2984, 2253, 1692, 1677, 1652, 1573, 1561, 1449, 1418, 1404, 1368, 1348, 1289, 1239, 1163, 1113, 1051, 924, 913, 906, 863, 797, 749, 728, 692, 661. ^1^H NMR (400 MHz, DMSO-*d*_6_) *δ* 9.60 (s, 1H, CH-Im), 8.04 (dm, *J* = 6.8 Hz, 2H, 2CH-Im), 5.78 (s, 2H, ImC*H*_2_CN), 4.60 (t, *J* = 6.5 Hz, 2H, C*H*_2_CH_2_CN), 3.28 (t, *J* = 6.5 Hz, 2H, CH_2_C*H*_2_CN). ^13^C NMR (101 MHz, DMSO) *δ* 138.3 (Im), 123.6 (Im), 123.5 (Im), 118.2 (CN), 115.1 (CN), 45.2 (Im*C*H_2_CN), 37.4 (*C*H_2_CH_2_CN), 19.0 (CH_2_*C*H_2_CN). Anal. calcd for C_8_H_9_ClN_4_: C, 48.86; H, 4.61; Cl, 18.03; N, 28.49%. Found: C, 48.74; H, 4.52; 17.87; N, 28.36%.

#### 1,3-Bis(2-cyanoethyl)-1*H*-imidazol-3-ium chloride 7

Yellow oil. IR (*ν*, cm^−1^): 3379, 3140, 3064, 2963, 2926, 2849, 1827, 2733, 2612, 2255, 1630, 1576, 1549, 1454, 1411, 1366, 1287, 907, 825, 755, 661. ^1^H NMR (400 MHz, DMSO-*d*_6_) *δ* 9.31 (s, 1H, CH-Im), 7.90 (s, 1H, CH-Im), 7.71 (s, 1H, CH-Im), 4.55 (t, *J* = 6.5 Hz, 2H, C*H*_2_CH_2_CN), 3.83 (t, *J* = 6.1 Hz, 2H, C*H*_2_CH_2_CN), 3.29 (t, *J* = 6.5 Hz, 2H, CH_2_C*H*_2_CN), 3.07 (t, *J* = 6.1 Hz, 2H, CH_2_C*H*_2_CN). ^13^C NMR (101 MHz, DMSO) *δ* 136.2 (Im), 122.3 (Im), 120.7 (Im), 118.9 (CN), 118.3 (CN), 44.4 (*C*H_2_CH_2_CN), 40.0 (*C*H_2_CH_2_CN), 21.8 (CH_2_*C*H_2_CN), 19.2 (CH_2_*C*H_2_CN). Anal. calcd for C_9_H_11_ClN_4_: C, 51.31; H, 5.26; Cl, 16.83; N, 26.60%. Found: C, 51.39; H, 17.02; N, 26.79%.

### Typical procedure for the synthesis of tetrachloridoferrate(iii) 8–11

A suspension of imidazolium chlorides and FeCl_3_·6H_2_O was left to stand for 2 days at room temperature, without requiring any sort of stirring or mechanical agitation. After removing H_2_O, the product was dried under reduced pressure. An analytical sample was obtained by recrystallization from diethyl ether, ethanol, hexane–ethanol or ethyl acetate.

#### 3-(Cyanomethyl)-1-methyl-1*H*-imidazol-3-ium tetrachloridoferrate(iii) 8

Yield 45.3%. M.p. 42–43 °C (diethyl ether). FT-IR (*ν*, cm^−1^): 3156, 3139, 3105, 3083, 2989, 2970, 2952, 2900, 2359, 2259, 1662, 1649, 1611, 1556, 1455, 1410, 1363, 1349, 1193, 1169, 1107, 1076, 1056, 1027, 1003, 948, 920, 850, 819. Anal. calcd for C_6_H_8_Cl_4_FeN_3_: C, 22.53; H, 2.52; N, 13.14%. Found: C, 22.47; H, 2.55; N, 13.18%. HPLC-MS: calcd. for [M]^+^ 319.87; found 319.81.

#### 3-(Cyanomethyl)-1-vinyl-1*H*-imidazol-3-ium tetrachloridoferrate(iii) 9

Yield 64.4%. M.p. 85–87 °C (ethanol). IR (*ν*, cm^−1^): 3158, 3139, 3105, 3083, 2989, 2952, 2359, 1662, 1649, 1622, 1611, 1564, 1556, 1410, 1363, 1349, 1276, 1193, 1172, 1107, 1003, 948, 920, 848. Anal. calcd for C_7_H_8_Cl_4_FeN_3_: C, 25.34; H, 2.43; N, 12.66%. Found: C, 25.29; H, 2.41; N, 12.68%. HPLC-MS: calcd. for [M]^+^ 331.88; found 331.82.

#### 1-(2-Cyanoethyl)-3-(cyanomethyl)-1*H*-imidazol-3-ium tetrachloridoferrate(iii) 10

Yield 38.5%. M.p. 96–98 °C (hexane–ethanol). IR (*ν*, cm^−1^): 3336, 3152, 3132, 3091, 2988, 2971, 2953, 2259, 1589, 1574, 1555, 1518, 1478, 1455, 1414, 1344, 1314, 1286, 1226, 1207, 1162, 1109, 1076, 1055, 1029, 1005, 921, 912, 818, 749, 734, 693, 659. Anal. calcd for C_8_H_9_Cl_4_FeN_4_: C, 26.78; H, 2.53; N, 15.61%. Found: C, 26.72; H, 2.49; N, 15.69%. HPLC-MS: calcd. for [M]^+^ 358.89; found 358.84.

#### 1,3-Bis(2-cyanoethyl)-1*H*-imidazol-3-ium tetrachloridoferrate(iii) 11

Yield 31.2%. M.p. 114–116 °C (ethyl acetate). IR (*ν*, cm^−1^): 3281, 3143, 3115, 3086, 2963, 2932, 2253, 1608, 1576, 1561, 1545, 1452, 1436, 1400, 1360, 1329, 1306, 1292, 1283, 1230, 1189, 1156, 1144, 1106, 1078, 1041, 1018, 1003, 910, 858, 847, 827, 751. Anal. calcd for C_9_H_11_Cl_4_FeN_4_: C, 28.99; H, 2.97; N, 15.03%. Found: C, 28.92; H, 2.99; N, 15.10%. HPLC-MS: calcd. for [M]^+^ 372.90; found 372.81.

### X-ray crystallography

X-ray data sets for 9 and 10 were collected on an Oxford Xcalibur diffractometer equipped with a CCD detector and graphite-monochromatized MoKα radiation (*λ* = 0.71073 Å) at room temperature 20 °C. The structures were solved by direct methods and refined by full-matrix least-squares on weighted *F*^2^ values for all reflections using the SHELX suite of programs.^60^ All non-H atoms in the compounds were refined with anisotropic displacement parameters. The positions of the hydrogen atoms in the structures were located on difference Fourier maps and refined isotropically. Crystallographic data and structure refinements of 9 and 10 are summarized in Table S1, selected bond distances are given in Table S2, and hydrogen bonds geometry in Table S3. The figures were drawn using the Mercury program. CCDC reference numbers for the structures are 2473775 (9) and 2473776 (10).

Crystal data for 9 at 293(2) K: C_7_H_8_Cl_4_FeN_3_, *M*_r_ = 331.84 g mol^−1^, 0.35 × 0.20 × 0.07 mm, triclinic, *P*1̄, *a* = 6.6339(6), *b* = 9.2678(9), *c* = 11.7121(10) Å, *α* = 104.408(8), *β* = 92.424(7), *γ* = 107.627(8)°, *V* = 659.26(11) Å^3^, *Z* = 2, 4011 reflections collected, 2435 independent (*R*_int_ = 0.0215), *R*_1_ = 0.0382 and w*R*_2_ = 0.0973 [*I* > 2 sigma(*I*)], *R*_1_ = 0.0471 and w*R*_2_ = 0.1048 (all data).

Crystal data for 10 at 293(2) K: C_8_H_9_Cl_4_FeN_4_, *M*_r_ = 358.81 g mol^−1^, 0.33 × 0.21 × 0.07 mm, monoclinic, *P*2_1_/*c*, *a* = 9.8765(5), *b* = 12.2383(4), *c* = 12.5215(6) Å, *β* = 108.106(5)°, *V* = 1438.55(12) Å^3^, *Z* = 4, 5393 reflections collected, 2640 independent (*R*_int_ = 0.0242), *R*_1_ = 0.0331 and w*R*_2_ = 0.0812 [*I* > 2 sigma(*I*)], *R*_1_ = 0.0463 and w*R*_2_ = 0.0901 (all data).

### Method for ^1^O_2_ generation activity of the studied compounds

The detection of the ^1^O_2_ generated in solution in the presence of the photocatalysts was carried out through electron spin resonance (ESR) spectroscopy in order to confirm the activity of the photocatalysts. An ESR Spectrometer device (MiniScope MS 300, Magnettech GmbH, Berlin, Germany) was used at a microwave power of 0.1 mW. The typical instrumental settings were: *B*_0_-field 336 mT, sweep width 10 mT, sweep time = 60 s, and a modulation amplitude of 0.2 mT. Samples of 5 mL volume of aqueous suspensions (10 mmol L^−1^) at the room temperature containing the photocatalyst and 40 mmol L^−1^ of 2,2,6,6-tetramethyl-4-piperidinol (TMP–OH) were exposed to UVA (*λ* = 365 nm, from a LightingcureTM UV spot light source, model LC-8, from Hamamatsu Photonics, Germany; photon flow: 9.57 × 10^−8^ einstein s^−1^, irradiation depth: 1.2 cm, irradiated surface: 5.72 cm^2^). During illumination, the suspensions were equilibrated with oxygen at atmospheric pressure. In order to record the ESR traces, 50 µL aliquots of illuminated suspensions were transferred into glass capillary tubes (0.7 mm ID, 0.87 mm OD, sample height of about 40 mm) and sealed on both ends with Cha-seal (Chase Scientific Glass, Rockwood, TN, USA). Furthermore, they were placed into quartz glass tubes (inner diameter of 4 mm, Magnettech GmbH, Berlin, Germany). Continuous wave X band ESR-Spectra of TEMPOL, formed from TMP–OH, were acquired at room temperature. An internal manganese standard (Mn^2+^ in ZnS) was measured simultaneously with the samples. The experimental procedure is described in detail in our early papers.^57,58^

### Proliferation assays

A panel of human cancer cell lines, including Capan-1, HCT-116, NCI–H460, LN-229, U87 MG, U138 MG, HL-60, K-562, Z-138, and DND-41, was used for cytotoxicity evaluation. Cells were maintained in appropriate culture media supplemented with 10% fetal bovine serum under standard conditions. Test compounds were prepared in dimethyl sulfoxide (DMSO) and diluted to the desired concentrations immediately prior to use.

Adherent cell lines (Capan-1, HCT-116, NCI-H460, LN-229, U87 MG, and U138 MG) were seeded in 384-well plates at densities ranging from 500 to 1500 cells per well, depending on growth characteristics. Suspension cell lines (HL-60, K-562, Z-138, and DND-41) were plated at densities between 2500 and 5500 cells per well. After overnight incubation, cells were exposed to seven serial dilutions of the test compounds (100 to 0.006 µM). Following 72 hours of treatment, cell viability was determined using the CellTiter 96® AQueous One Solution (MTS) assay according to the manufacturer's protocol. Absorbance was measured at 490 nm, and IC_50_ values were calculated from optical density readings. Each compound was tested in at least two independent experiments.

### PBMC viability

A buffy coat sample obtained as leftover material from a healthy blood donor was provided by the Blood Transfusion Center in Leuven (Belgium) through the Red Cross Flanders biobank (reference number M20210510A). Peripheral blood mononuclear cells (PBMCs) were isolated *via* density gradient centrifugation using Lymphoprep and subsequently cultured in RPMI medium supplemented with 10% fetal bovine serum. PBMCs were seeded at a density of 28 000 cells per well in 384-well plates and treated with test compounds at concentrations ranging from 100 to 0.006 µM. After a 72 hours incubation period, cell viability was evaluated using the MTS assay, with absorbance measured at 490 nm to calculate IC_50_ values from optical density readings.

### SQUID magnetometry

Magnetic data for 9 and 10 were collected using a Quantum Design MPMS-XL SQUID magnetometer with the polycrystalline samples each immobilized in cylindrical PTFE capsules. Experimental data were collected in the temperature range 2.0–300 K at 0.1 and 1.0 T, and in the magnetic field range 0–7.0 T at 2.0 K. The data were corrected for the diamagnetic contributions of both the sample holder and the intrinsic contribution of 9 or 10, respectively (*χ*_m,dia_/10^−4^ cm^3^ mol^−1^ = −1.66 (9), −1.79 (10)).

## Conclusion

In this work, a new series of imidazole-based tetrachloridoferrate(iii) ionic liquids was synthesized and comprehensively investigated for their structural, magnetic, photochemical, and biological properties. Single-crystal X-ray diffraction of selected representatives revealed a characteristic supramolecular organization governed by non-covalent interactions and anisotropic charge distribution, providing valuable insight into the factors that determine their stability in the liquid state and during reaction processes. The synthesized imidazole-based tetrachloridoferrate salts demonstrated selective cytotoxicity toward the LN229 glioblastoma cell line with IC_50_ values ranging from 1.05 to 3.40 µM, which is comparable to that of the reference drug etoposide. No significant activity was observed against other solid tumour or haematological cancer cell lines. The compounds were also inactive against U87 MG and U138 MG glioblastoma models. Importantly, all compounds exhibited markedly reduced cytotoxicity towards PBMCs with IC_50_ values in the range of 10.3–59.8 µM, with compound 8 displaying the highest selectivity index (SI = 44), underscoring its potential as a targeted anticancer candidate. Additionally, ESR experiments showed efficient formation of singlet molecular oxygen (^1^O_2_) by the compounds in aqueous suspension. This reactive oxygen species plays a critical role in photochemical processes and is a key mediator of photodynamic effects in biological systems. SQUID magnetometry revealed the presence of high-spin Fe^3+^ centers within compounds 9 and 10, which engage in weak antiferromagnetic exchange interactions, likely along chains in the frozen ionic liquid state. These weak interactions influence the magnetic behavior only at temperatures below 50 K.

Collectively, the obtained results demonstrate that imidazole-based tetrachloridoferrate(iii) salts represent a promising structural platform for the development of selective anti-glioblastoma agents. Their unique combination of photochemical activity, redox properties, and selective cytotoxicity warrants further mechanistic studies and optimization to advance this class of compounds toward potential biomedical applications.

## Author contributions

Conceptualization: M. N., F. M. Funding acquisition: M. N., S. N., F. M. Investigation: E. S., N. S., M. N., S. G. B., V. Ch. K., J. van L., P. K., L. P., D. D., S. de J. Methodology: E. P., P. K., L. P., S. de J. Project administration: M. N., F. M. Validation: N. S., S. G. B. Visualization: N. S., S. G. B. Writing – original draft: M. N., N. S., S. G. B., F. M. Writing – review & editing: M. N., N. S., S. G. B.

## Conflicts of interest

The authors declare no conflicts of interest.

## Supplementary Material

RA-OLF-D6RA05375K-s001

RA-OLF-D6RA05375K-s002

## Data Availability

The data supporting the findings of this study are available within the article and its supplementary information (SI). Supplementary information: additional crystallographic and structural data (Tables S1–S3), ^1^H, ^13^C NMR, DEPT and FT-IR spectra of compounds 4–7 (Fig. S1–S15), as well as FT-IR and mass spectra of compounds 8–11 (Fig. S16–S29). See DOI: https://doi.org/10.1039/d6ra05375k. CCDC 2473775 (9) and 2473776 (10) contain the supplementary crystallographic data for this paper.^61*a*,*b*^
