# Supplementary material for: Imidazole-based tetrachloridoferrate(iii) magnetic ionic liquids: structural insights, photosensitizing potential, and selective anti-glioblastoma activity
Source: RSC Adv. 2026 Aug 21;16(41):49168–77. doi: 10.1039/d6ra05375k (PMC13494830; doi:10.1039/d6ra05375k)
Supplement: Supplementary file 1 [file RA-016-D6RA05375K-s001.pdf]

Supplementary Information (SI) for RSC Advances.
This journal is © The Royal Society of Chemistry 2026

Supporting information

# Imidazole-based tetrachloridoferrate(III) magnetic ionic liquids: Structural insights, photosensitizing potential, and selective anti-glioblastoma activity

*Eugenia Stingaci,* [a] *Natalia Sucman,**[a] *Mariana Neamtu,*[b] *Svetlana G. Baca,*[c] *Victor Ch. Kravtsov,*[c] *Jan van Leusen,*[d] *Paul Kögerler,*[d] *Leentje Persoons,*[e] *Dirk Daelemans,*[e] *S. De Jonghe,*[f] *and Fliur Z. Macaev**[a]

[a]Laboratory of Organic Synthesis, Institute of Chemistry, Moldova State University, 3 Academiei str., MD-2028 Chisinau, R. Moldova.

[b]Department of Exact and Natural Sciences Institute of Interdisciplinary Research „Alexandru Ioan Cuza" University. Bv. Carol I, no. 11, 700506 Iași, Romania

[c]Laboratory of Physical Methods of Solid State Investigation "Tadeusz Malinowski" Institute of Applied Physics, Moldova State University, 5 Academiei str., MD-2028 Chisinau, R. Moldova

[d]Institute of Inorganic Chemistry, RWTH Aachen University, Landoltweg 1, D-52074 Aachen, Germany

[e]Molecular Genetics and Therapeutics in Virology and Oncology Research Group, Rega Institute for Medical Research, Department of Microbiology, Immunology and Transplantation, KU Leuven, Herestraat 49, P.O. Box 1048, B-3000 Leuven, Belgium

[f]Molecular, Structural and Translational Virology Research Group, Rega Institute for Medical Research, Department of Microbiology, Immunology and Transplantation, KU Leuven, Herestraat 49, P.O. Box 1049, B-3000 Leuven, Belgium

**Corresponding authors:** *E-mail: fliur.macaev@sti.usm.md; natalia_sucman@yahoo.com*

**Table S1.** Crystal data and details of structural determinations for **9** and **10**

| | **9** | **10** |
|---|---|---|
| Formula | $C_7H_8Cl_4FeN_3$ | $C_8H_9Cl_4FeN_4$ |
| $M_r$ / g mol$^{-1}$ | 331.81 | 358.84 |
| $T$ / K | 293(2) | 293(2) |
| Cryst system | triclinic | monoclinic |
| Space group | $P$-1 | $P2_1/c$ |
| $a$ / Å | 6.6339(6) | 9.8765(5) |
| $b$ / Å<br>$c$ / Å | 9.2678(9)<br>11.7121(10) | 12.2383(4)<br>12.5215(6) |
| $\alpha$ / ° | 104.408(8) | 90 |
| $\beta$ / ° | 92.424(7) | 108.106(5) |
| $\gamma$ / ° | 107.627(8) | 90 |
| $V$, Å$^3$ | 659.26(11) | 1438.55(12) |
| $Z$ | 2 | 4 |
| $D_{calcd}$ / mg/m$^3$ | 1.672 | 1.657 |
| $\mu$ / mm$^{-1}$ | 1.925 | 1.773 |
| $F$(000) | 330 | 716 |
| Crystal size / mm$^3$ | 0.35 x 0.20 x 0.07 | 0.33 x 0.21 x 0.07 |
| Theta range for data collection, ° | 3.249 – 25.496 | 3.329 – 25.500 |
| Index ranges | $-8 \le h \le 7$,<br>$-11 \le k \le 11$,<br>$-14 \le l \le 12$ | $-11 \le h \le 11$,<br>$-13 \le k \le 14$,<br>$-15 \le l \le 11$ |
| Reflections collected / independent reflections | 4011/2435<br>($R_{int}$ = 0.0215) | 5393/2640<br>($R_{int}$ = 0.0242) |
| Completeness to theta / % ($\vartheta$=25.50) | 99.6 | 99.3 |
| Data/restrains/parameters | 2435 / 0 / 169 | 2640 / 0 / 191 |
| Goodness-of-fit on $F^2$ | 1.000 | 1.000 |
| Final $R_1$, $wR_2$ | $R_1$ = 0.0382,<br>$wR_2$ = 0.0973 | $R_1$ = 0.0331,<br>$wR_2$ = 0.0812 |
| $R$ indices (all data) | $R_1$ = = 0.0471,<br>$wR_2$ = 0.1048 | $R_1$ = = 0.0463,<br>$wR_2$ = 0.0901 |
| Extinction coefficient | 0.010(2) | 0.0042(8) |
| Largest diff. peak and hole, $e$·Å$^{-3}$ | 0.324, –0.323 | 0.432, –0.298 |
| CCDC | 2473775 | 2473776 |

**Table S2.** Selected bond distances (Å) in **9** and **10**

| **9** | | **10** | |
|---|---|---|---|
| Fe(1)–Cl(1) | 2.1810(11) | Fe(1)–Cl(4) | 2.1797(8) |
| Fe(1)–Cl(3) | 2.1813(11) | Fe(1)–Cl(3) | 2.1830(8) |
| Fe(1)–Cl(2) | 2.1926(10) | Fe(1)–Cl(1) | 2.1916(8) |
| Fe(1)–Cl(4) | 2.1971(10) | Fe(1)–Cl(2) | 2.2022(8) |

**Table S3.** Details of the hydrogen bonding interactions in **9** and **10**

| Contact D–H···A | Distance, Å | | | Angle | Symmetry |
|---|---|---|---|---|---|
| | D–H | H···A | D···A | DHA, ° | Transformations |
| **9** | | | | | |
| C(1)-H(1)...N(3) | 0.97(4) | 2.58(4) | 3.326(5) | 135(3) | *x*-1, *y*, *z* |
| C(1)-H(1)...N(3) | 0.97(4) | 2.51(3) | 3.252(5) | 134(3) | -*x*+2, -*y*+1, -*z*+2 |
| C(2)-H(2)...Cl(4) | 0.87(3) | 2.87(3) | 3.718(4) | 165(3) | *x*+1, *y*-1, *z* |
| C(3)-H(3)...Cl(2) | 0.93(3) | 2.86(4) | 3.790(4) | 171(3) | -*x*+1, -*y*+1, -*z*+1 |
| C(4)-H(4B)...Cl(2) | 0.95(4) | 2.89(4) | 3.544(4) | 128(3) | *x*, *y*-1, *z* |
| **10** | | | | | |
| C(4)-H(4B)...N(4) | 0.98(3) | 2.48(3) | 3.303(4) | 142(2) | -*x*+2, *y*-1/2, -*z*+1/2 |
| C(6)-H(6A)...Cl(1) | 0.93(3) | 2.80(3) | 3.619(3) | 147.4(2) | *x*, *y*, *z* |
| C(6)-H(6B)...Cl(4) | 0.99(3) | 2.88(3) | 3.756(3) | 149(2) | -*x*+1, *y*+1/2, -*z*+1/2 |
| C(7)-H(7A)...Cl(2) | 0.90(3) | 2.93(3) | 3.687(3) | 143(2) | -*x*+1, *y*+1/2, -*z*+1/2 |

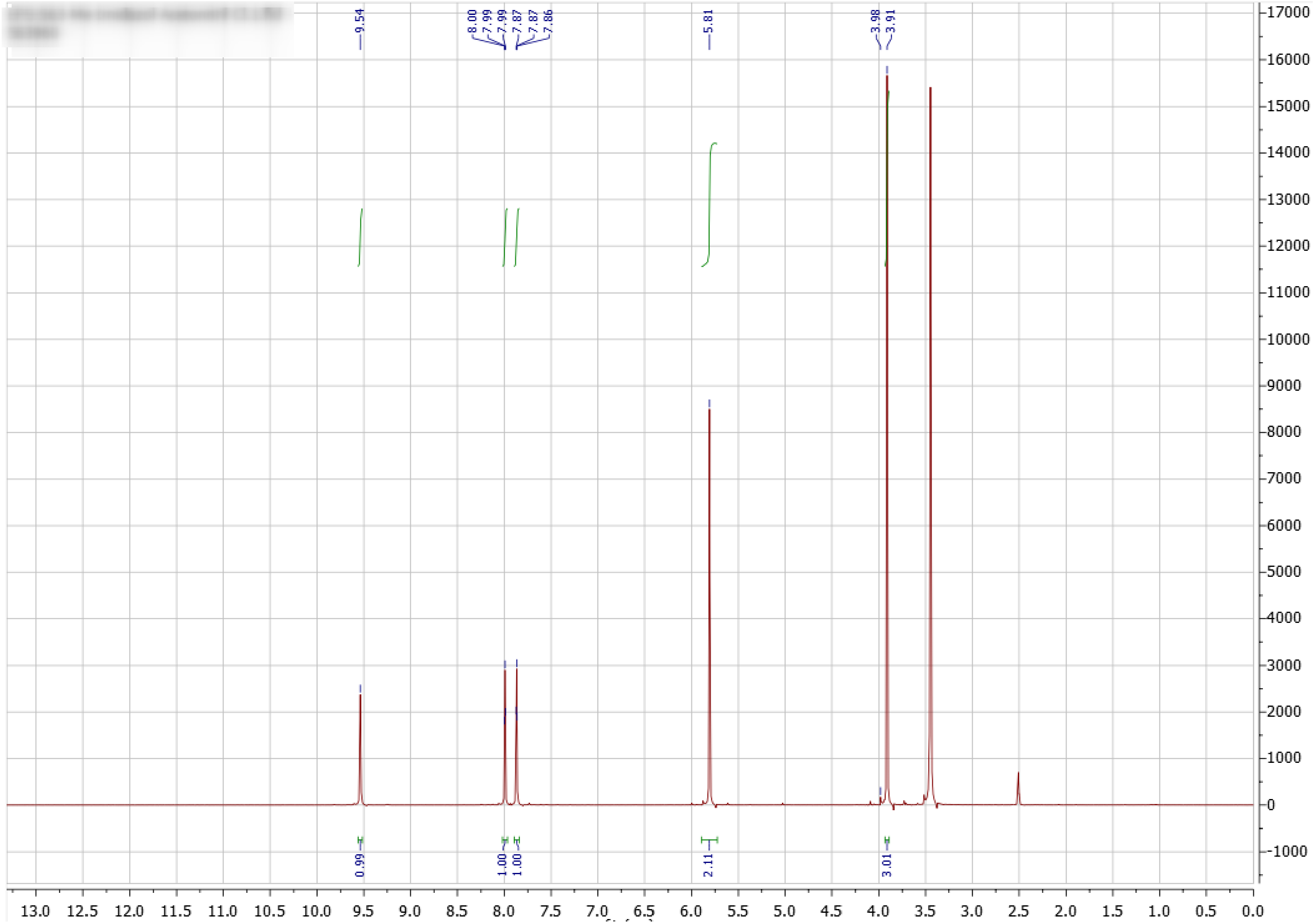


**Figure S1.** $^{1}$H-NMR spectra of 3-(cyanomethyl)-1-methyl-1*H*-imidazol-3-ium chloride (**4**).

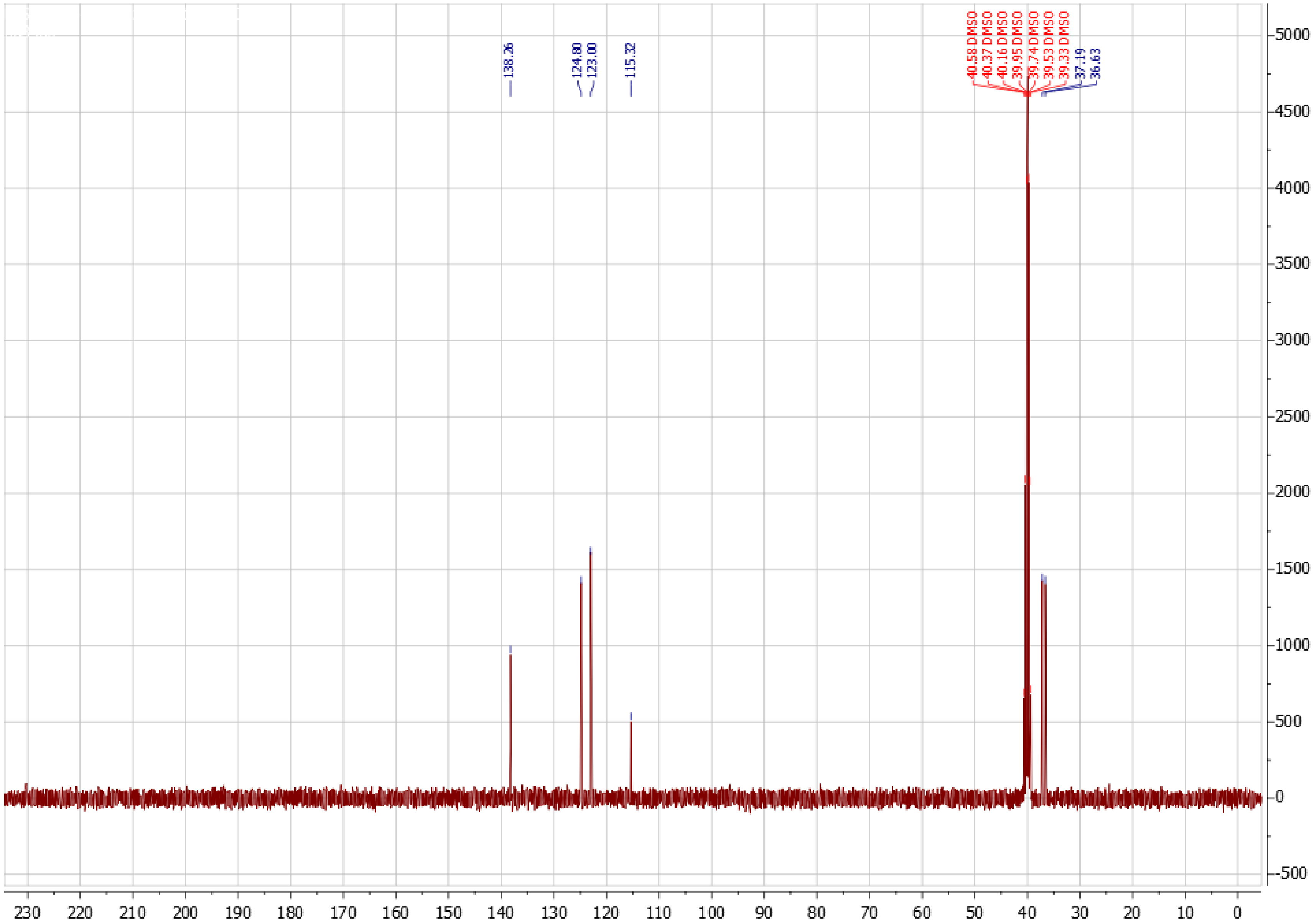


**Figure S2.** $^{13}$C-NMR spectra of 3-(cyanomethyl)-1-methyl-1*H*-imidazol-3-ium chloride (**4**).

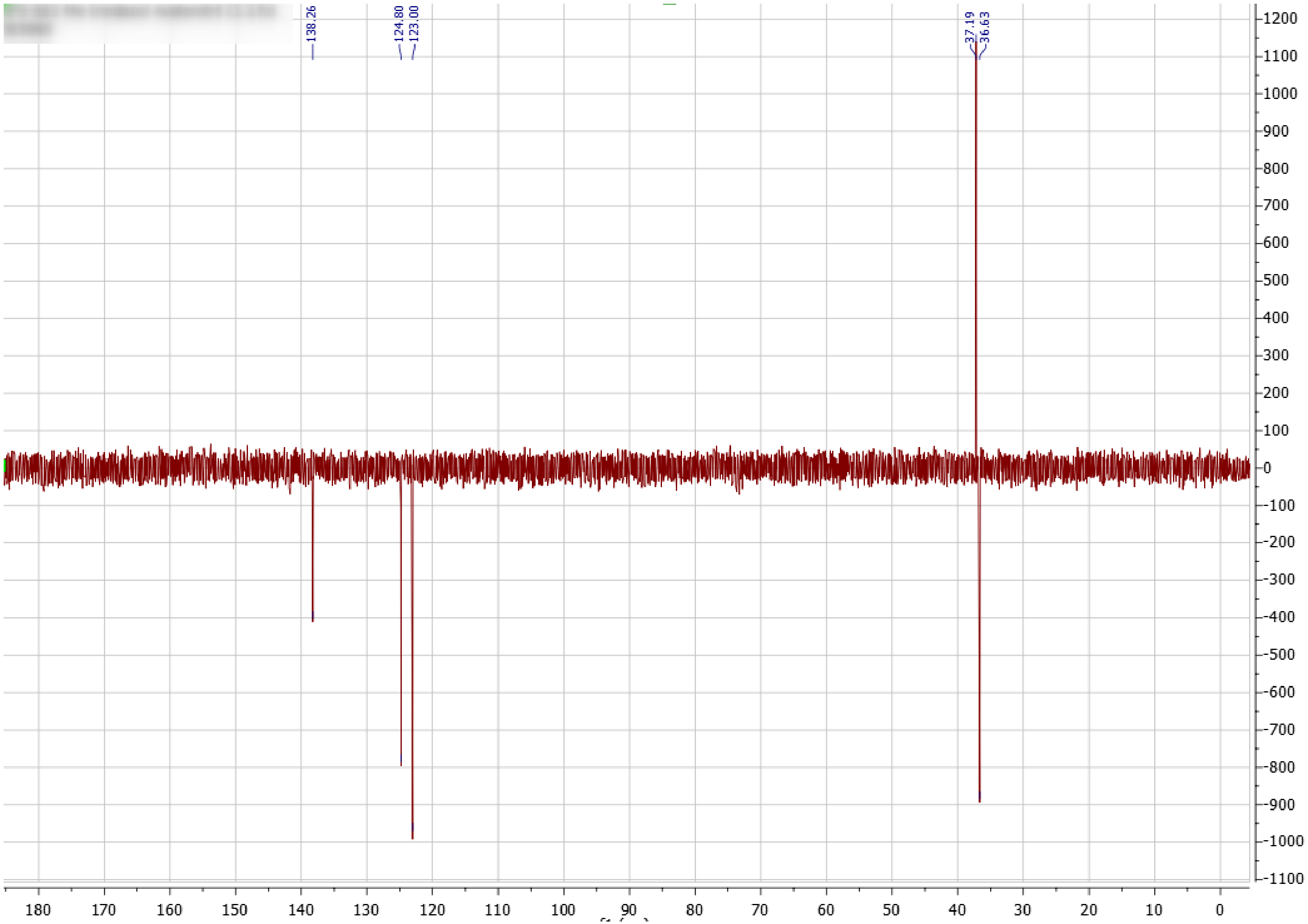


**Figure S3.** DEPT-NMR spectra of 3-(cyanomethyl)-1-methyl-1*H*-imidazol-3-ium chloride (**4**).

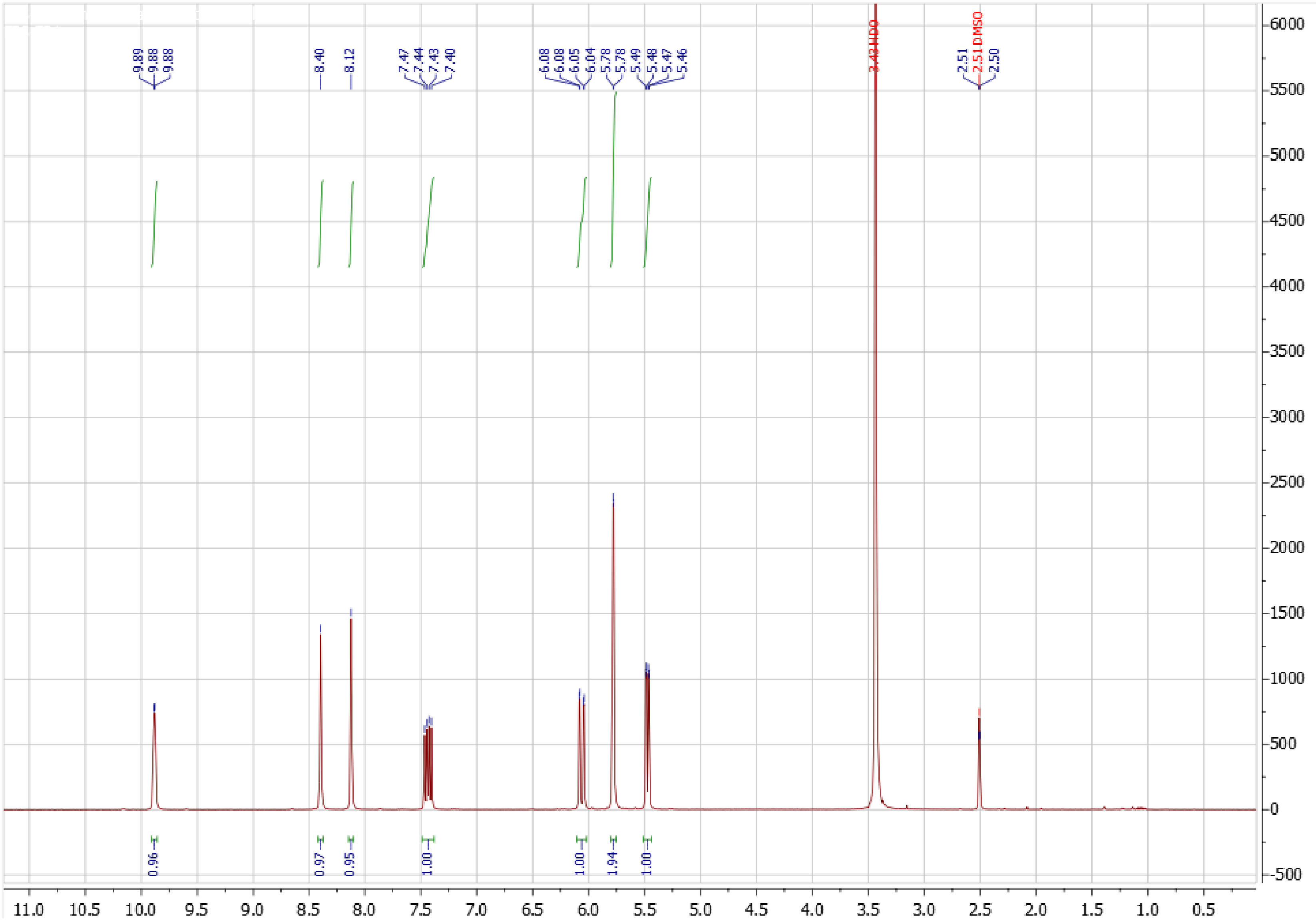


**Figure S4.** $^{1}$H-NMR spectra of 3-(cyanomethyl)-1-vinyl-1*H*-imidazol-3-ium chloride (**5**).

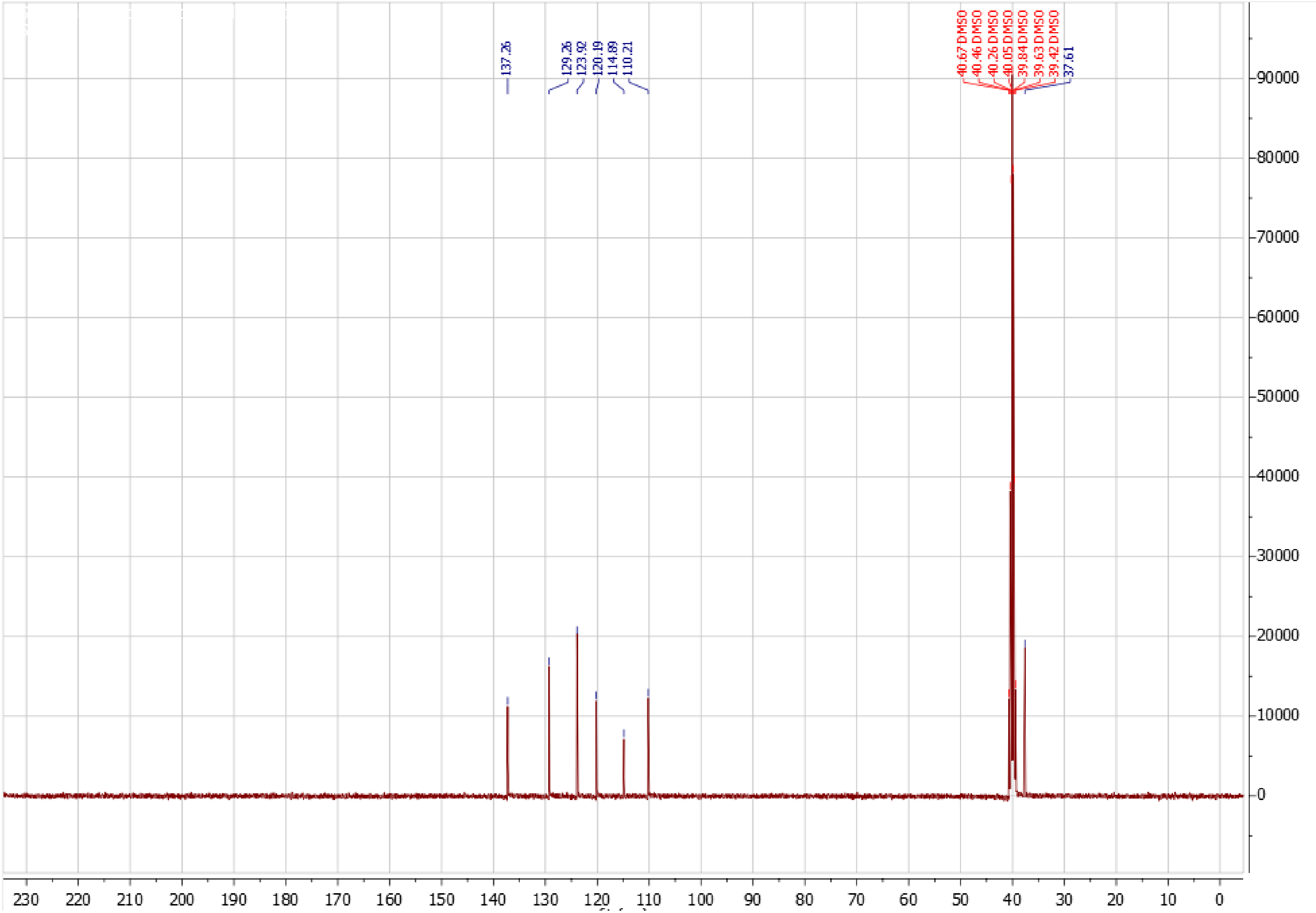


**Figure S5.** $^{13}$C-NMR spectra of 3-(cyanomethyl)-1-vinyl-1*H*-imidazol-3-ium chloride (**5**).

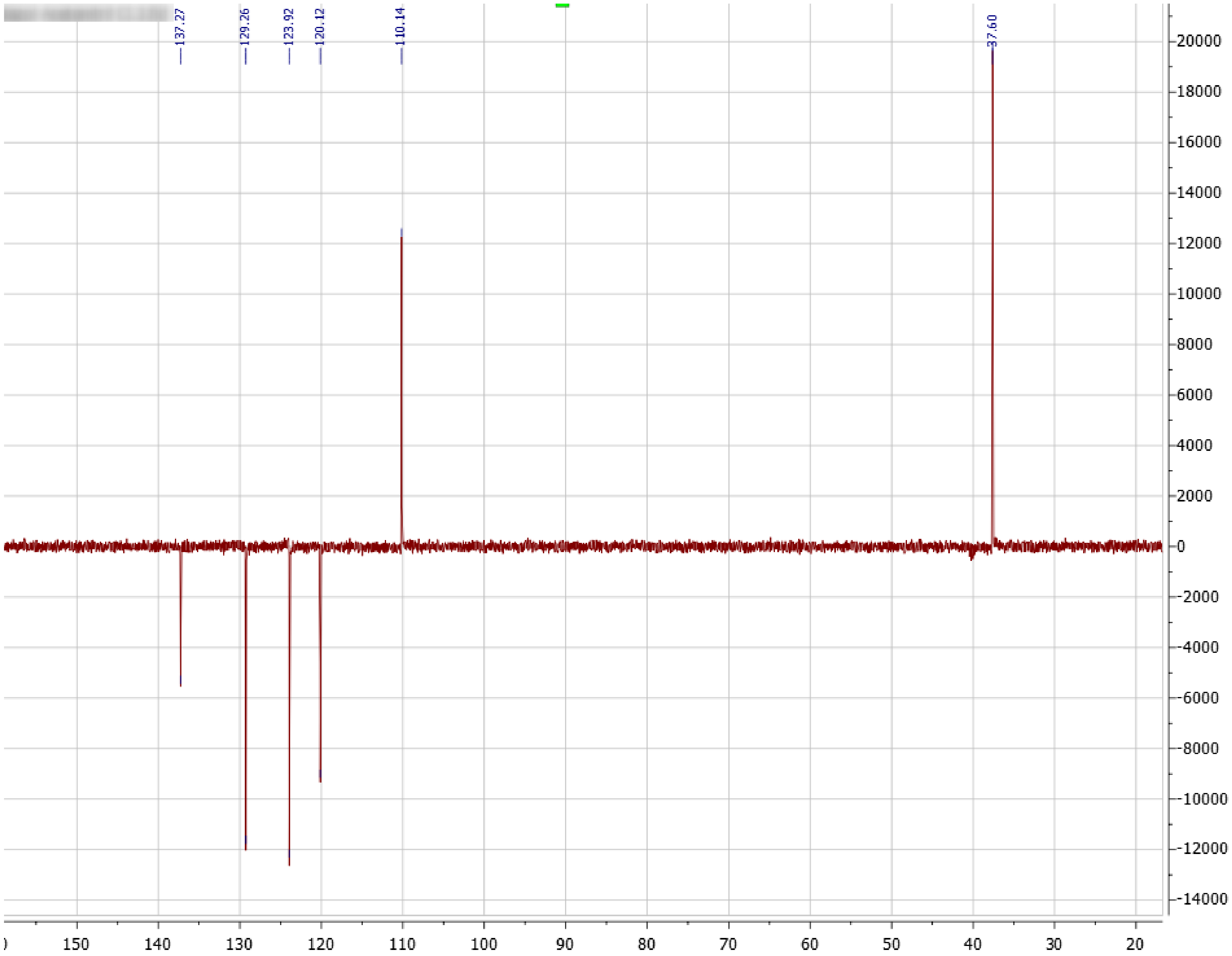


**Figure S6.** DEPT-NMR spectra of 3-(cyanomethyl)-1-vinyl-1*H*-imidazol-3-ium chloride (**5**).

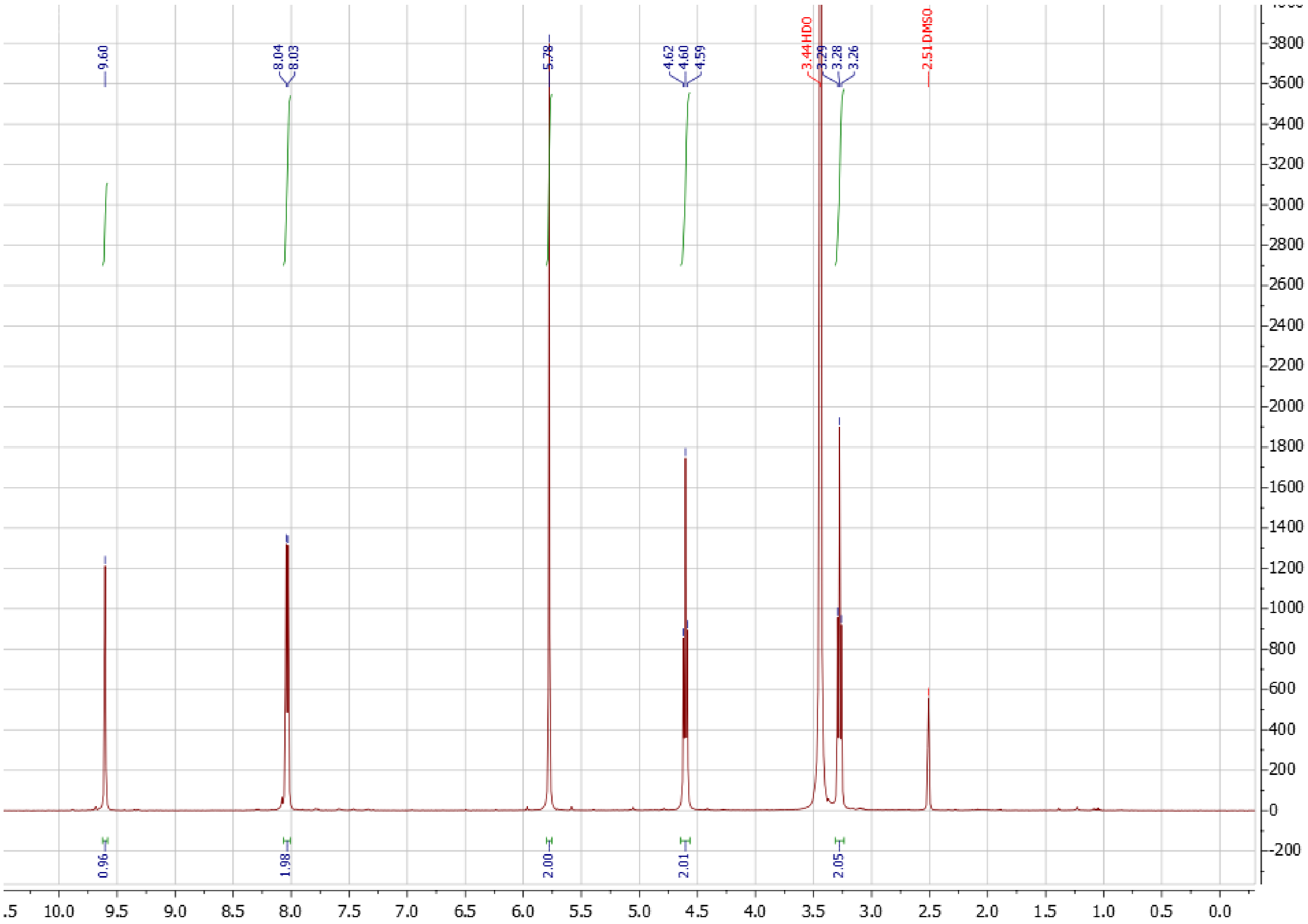

**Figure S7.** $^1$H-NMR spectra of 1-(2-cyanoethyl)-3-(cyanomethyl)-1*H*-imidazol-3-ium chloride (**6**).

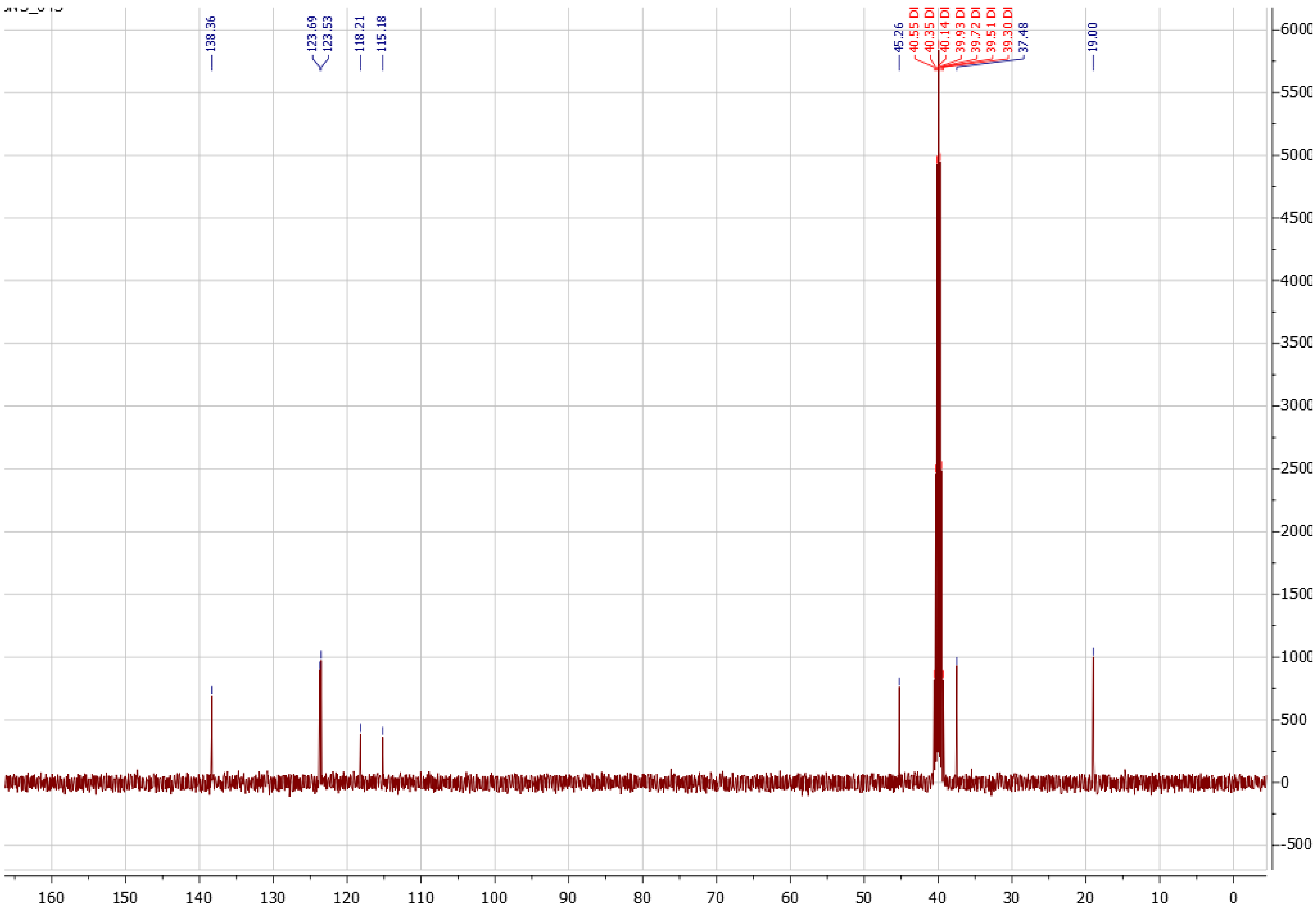


**Figure S8.** $^{13}$C-NMR spectra of 1-(2-cyanoethyl)-3-(cyanomethyl)-1*H*-imidazol-3-ium chloride (**6**).

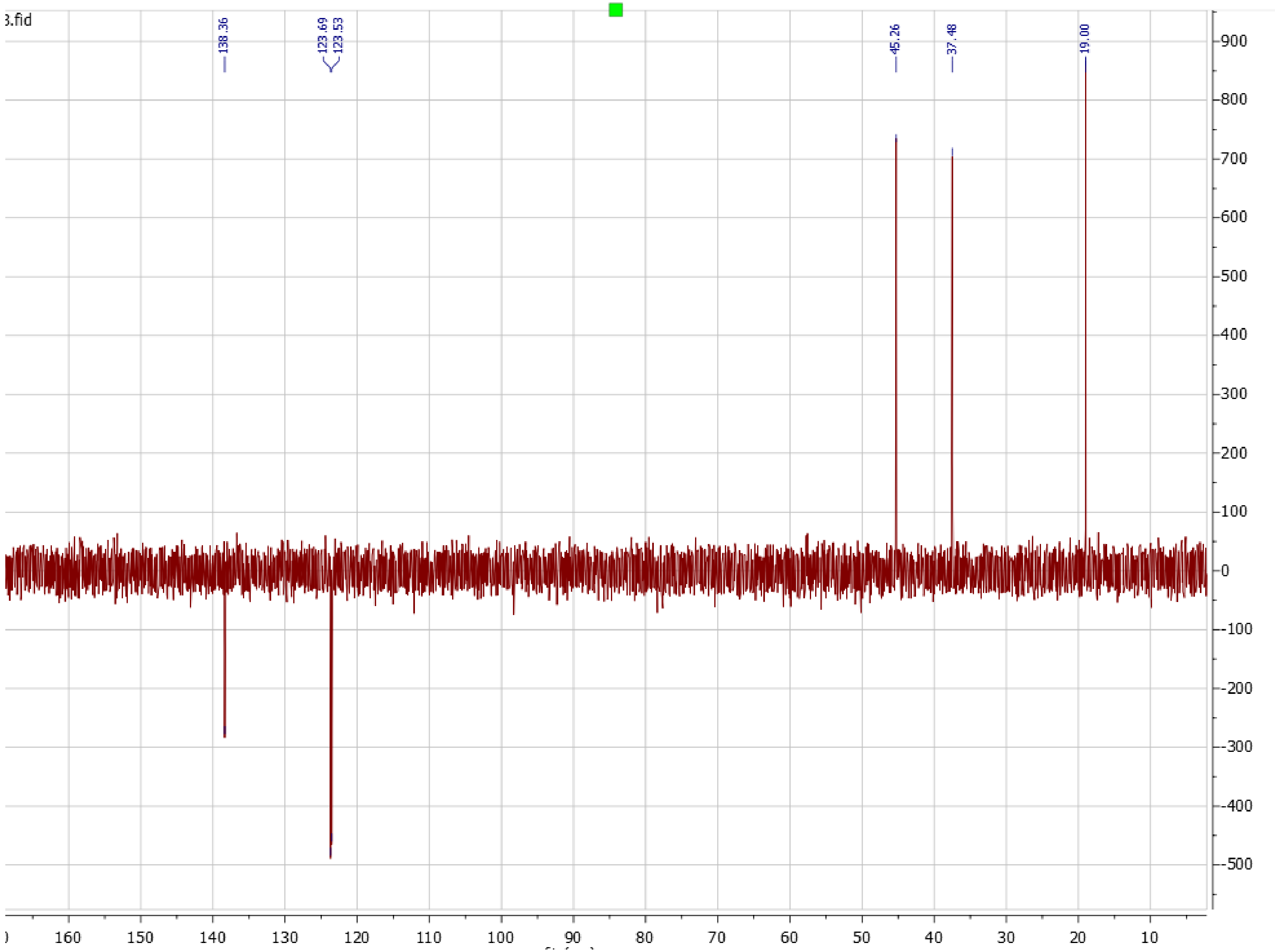


**Figure S9.** DEPT-NMR spectra of 1-(2-cyanoethyl)-3-(cyanomethyl)-1*H*-imidazol-3-ium chloride (**6**).

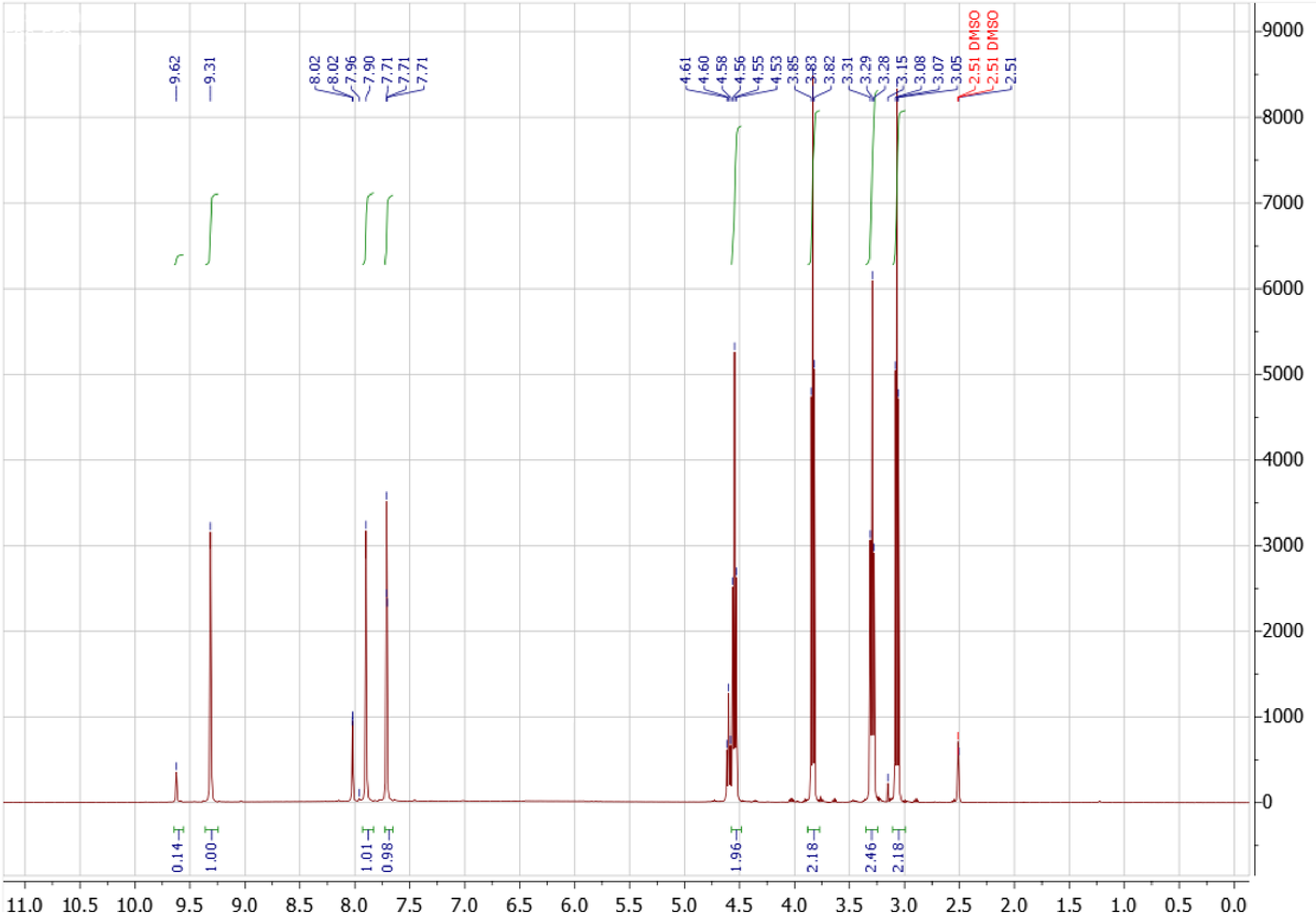


**Figure S10.** $^{1}$H-NMR spectra of 1,3-bis(2-cyanoethyl)-1*H*-imidazol-3-ium chloride (**7**).

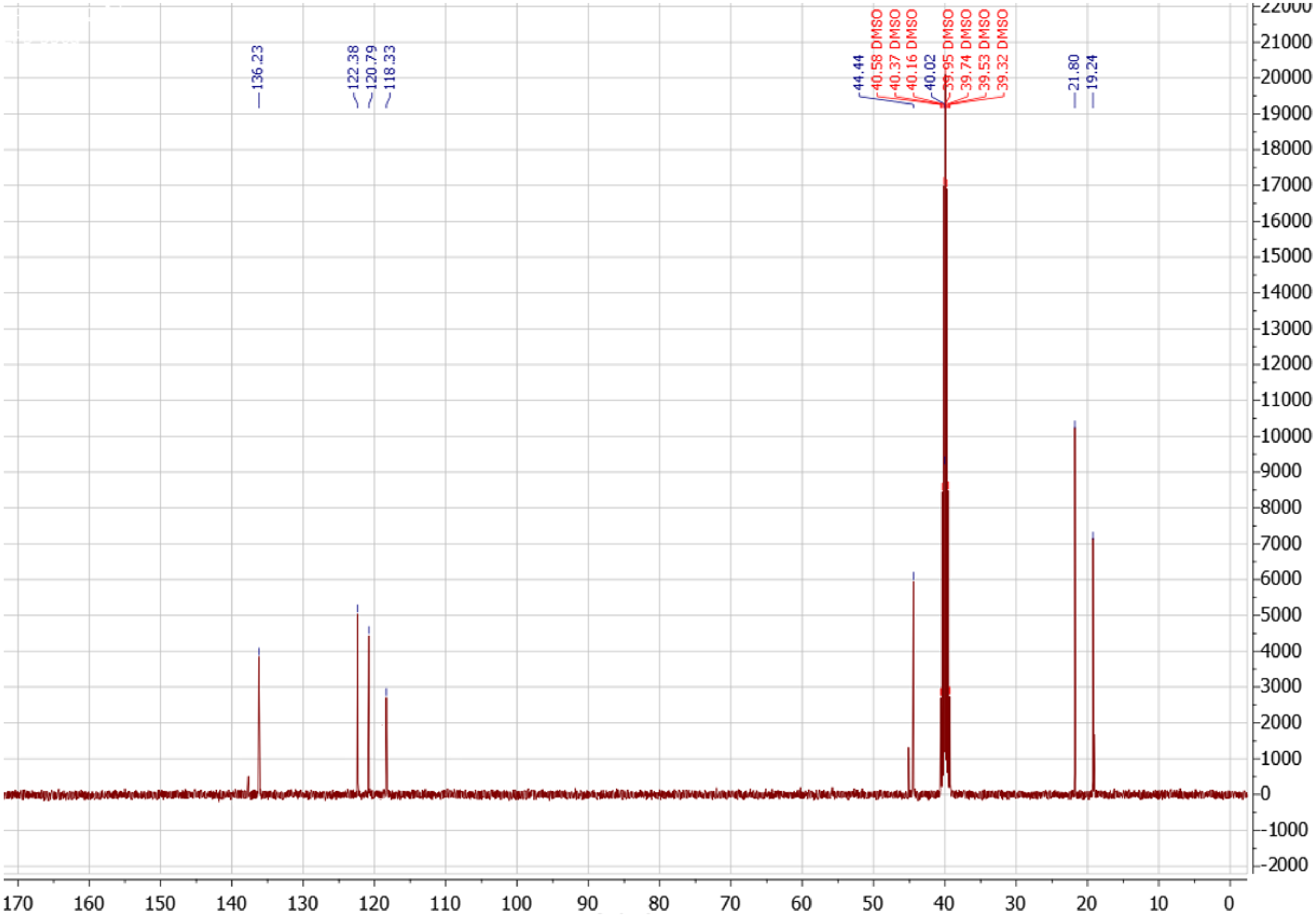


**Figure S11.** $^{13}$C-NMR spectra of 1,3-bis(2-cyanoethyl)-1*H*-imidazol-3-ium chloride (**7**).

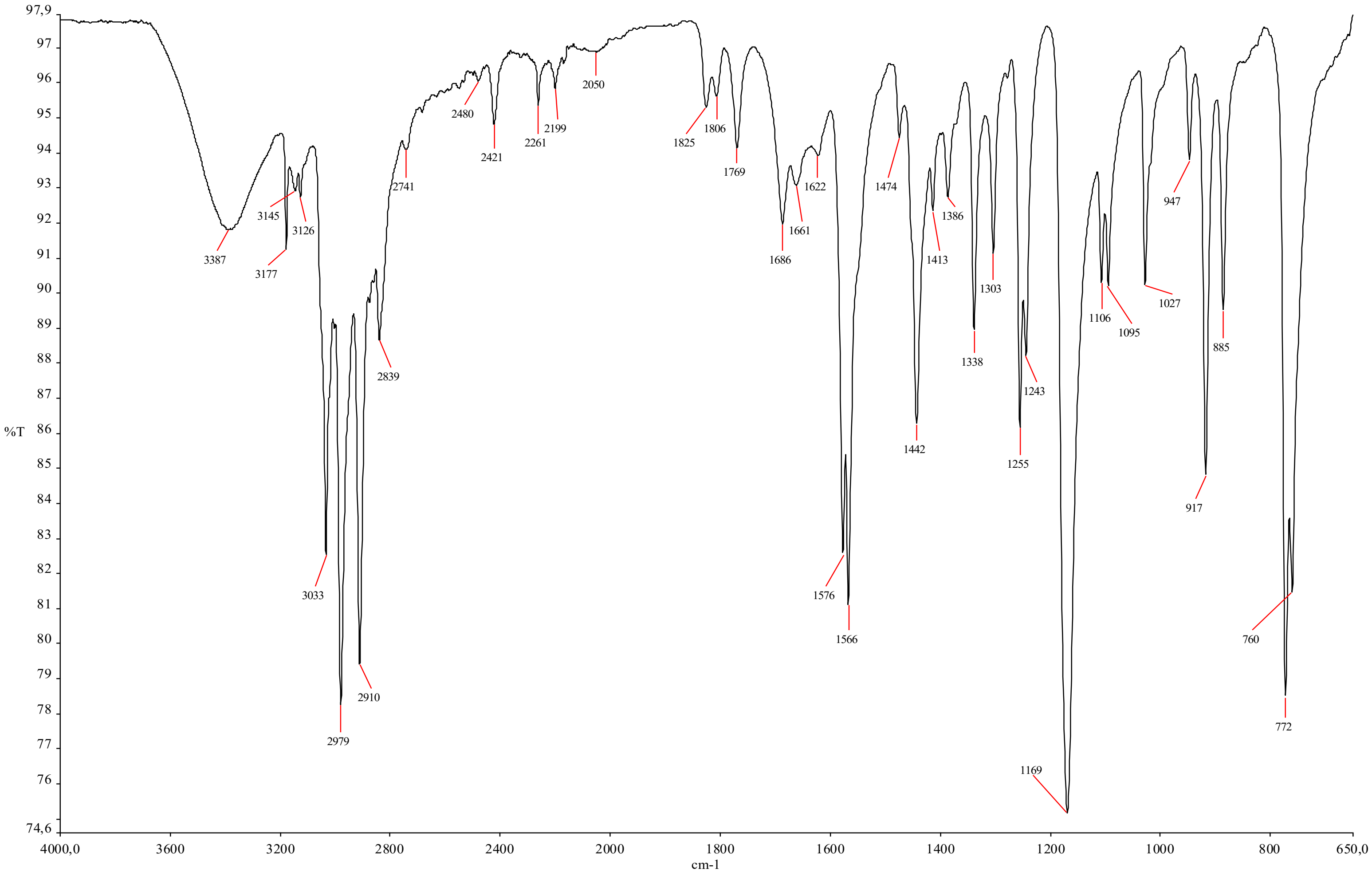


**Figure S12.** FTIR spectra of 3-(cyanomethyl)-1-methyl-1*H*-imidazol-3-ium chloride (**4**).

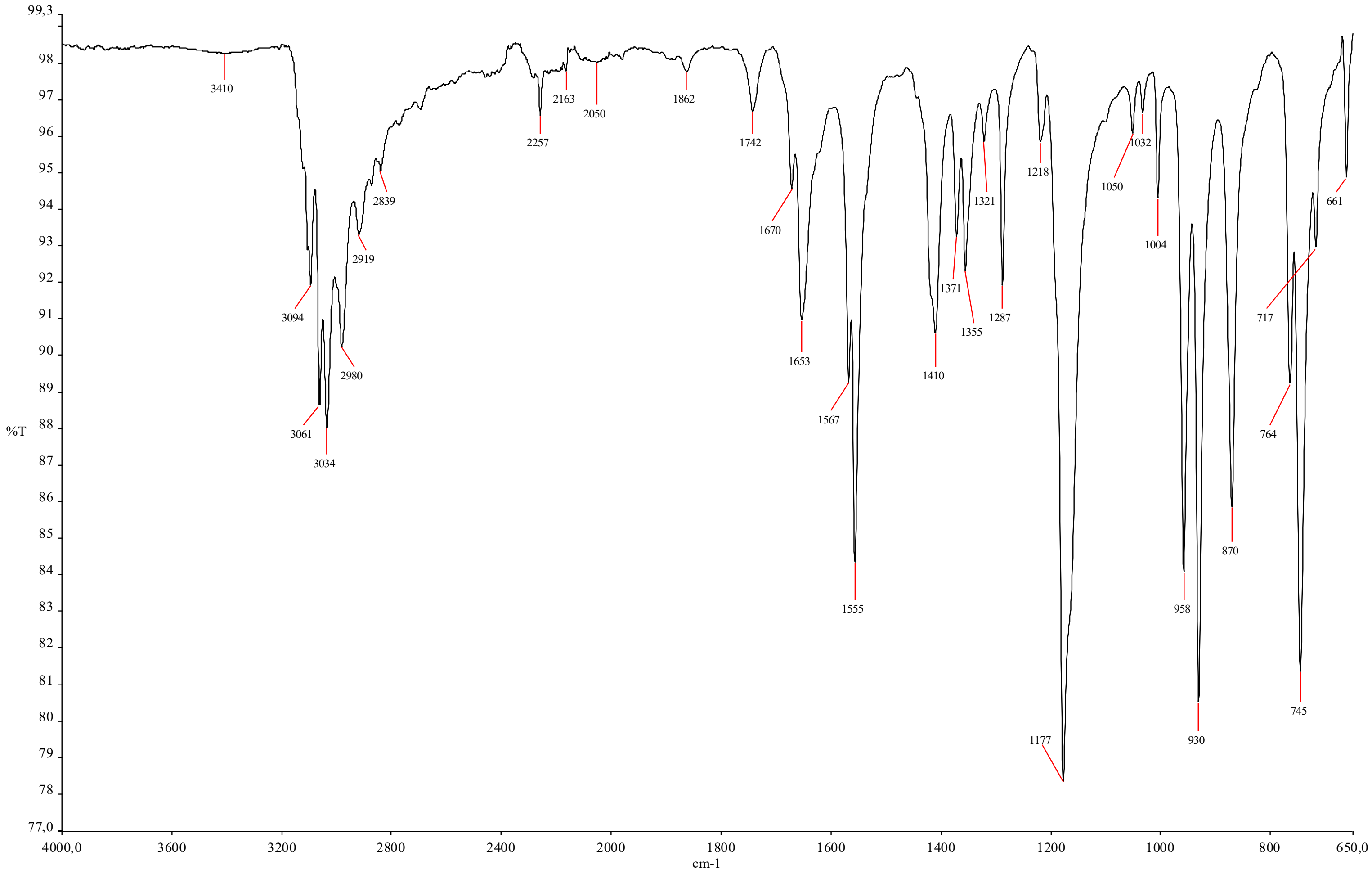


**Figure S13.** FTIR spectra of 3-(cyanomethyl)-1-vinyl-1*H*-imidazol-3-ium chloride (**5**).

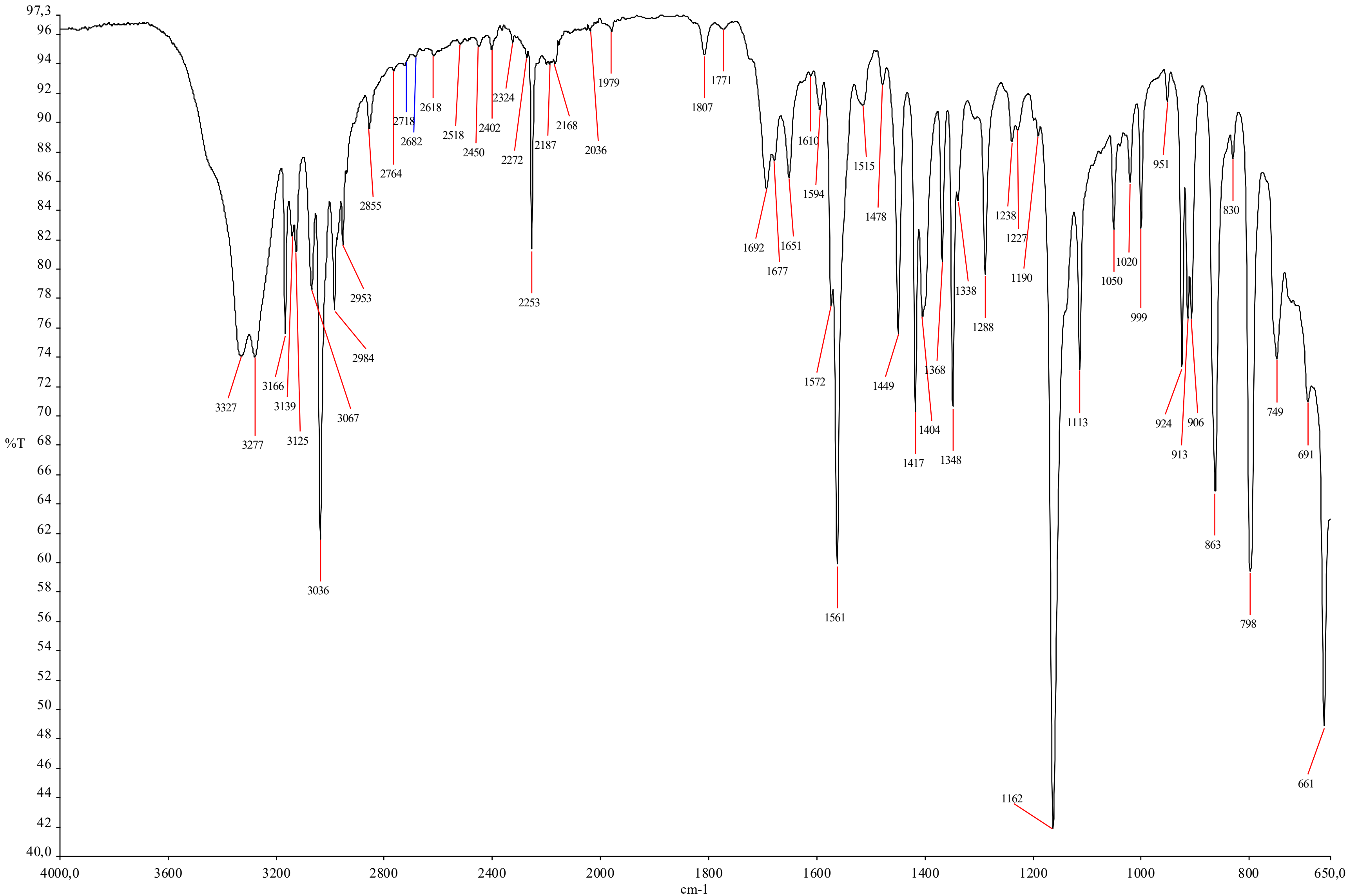


**Figure S14.** FTIR spectra for 1-(2-cyanoethyl)-3-(cyanomethyl)-1*H*-imidazol-3-ium chloride (**6**).

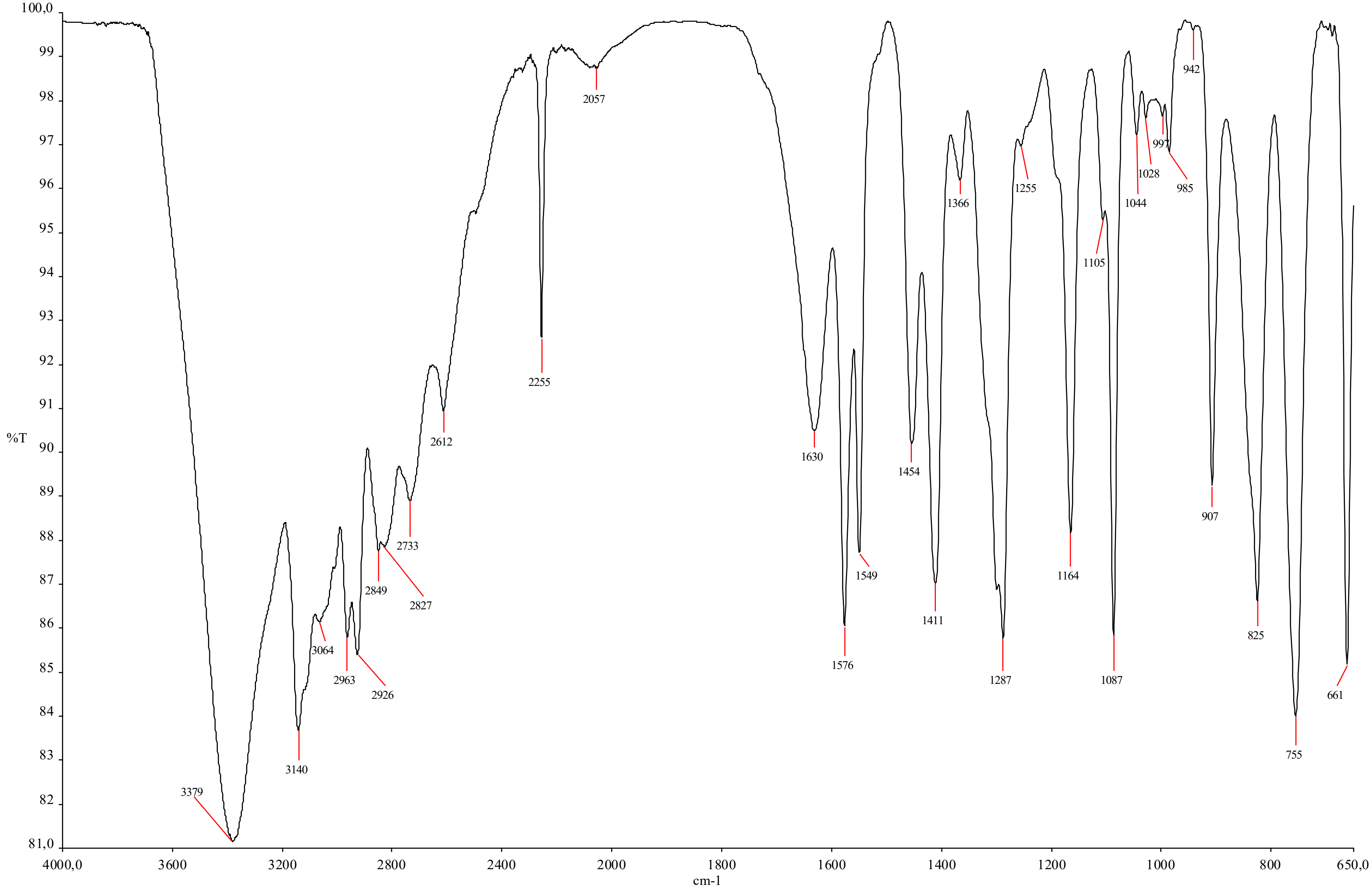


**Figure S15.** FTIR spectra of 1,3-bis(2-cyanoethyl)-1*H*-imidazol-3-ium chloride (**7**).

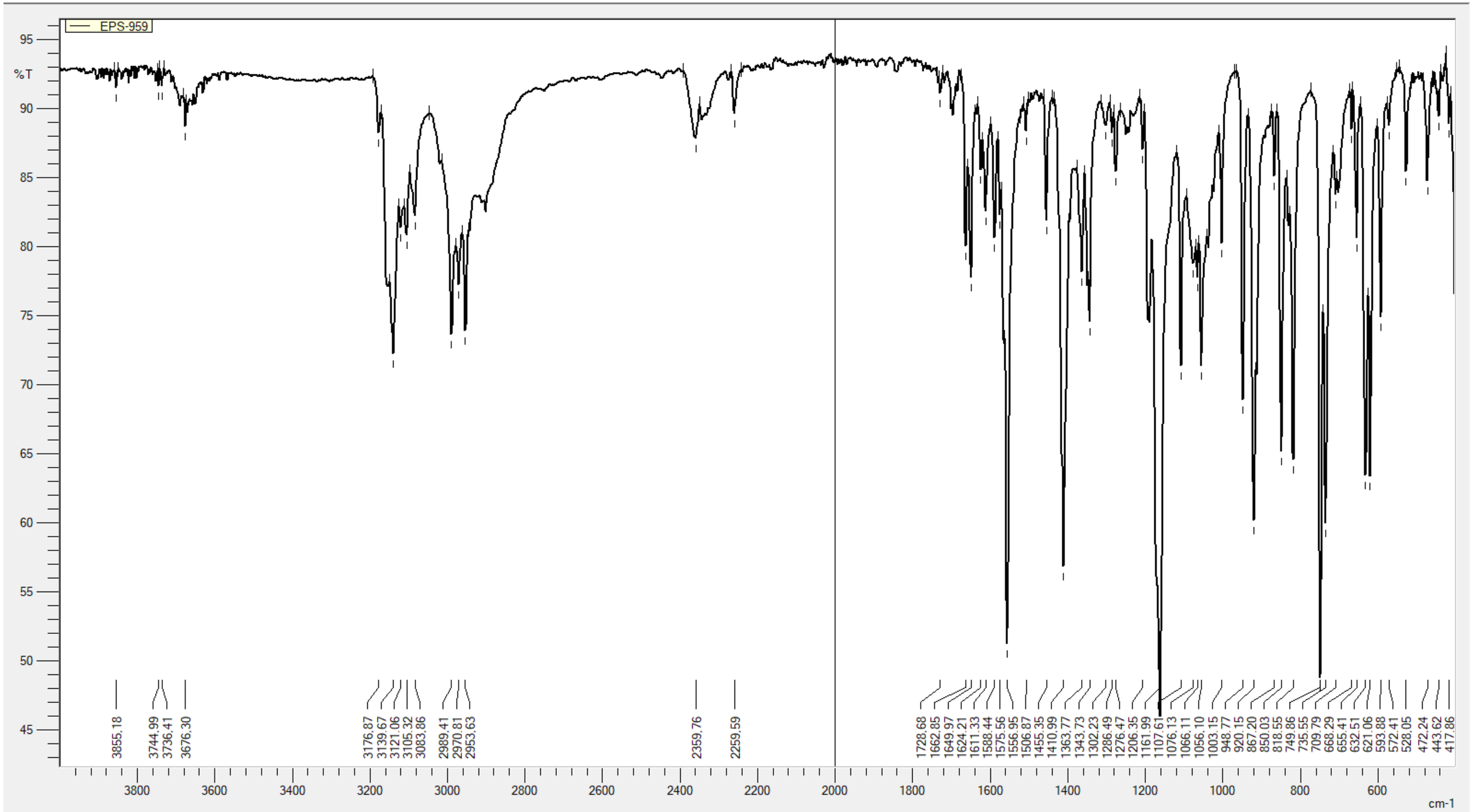


**Figure S16.** IR spectra of 3-(cyanomethyl)-1-methyl-1*H*-imidazol-3-ium tetrachloridoferrate(III) (**8**).

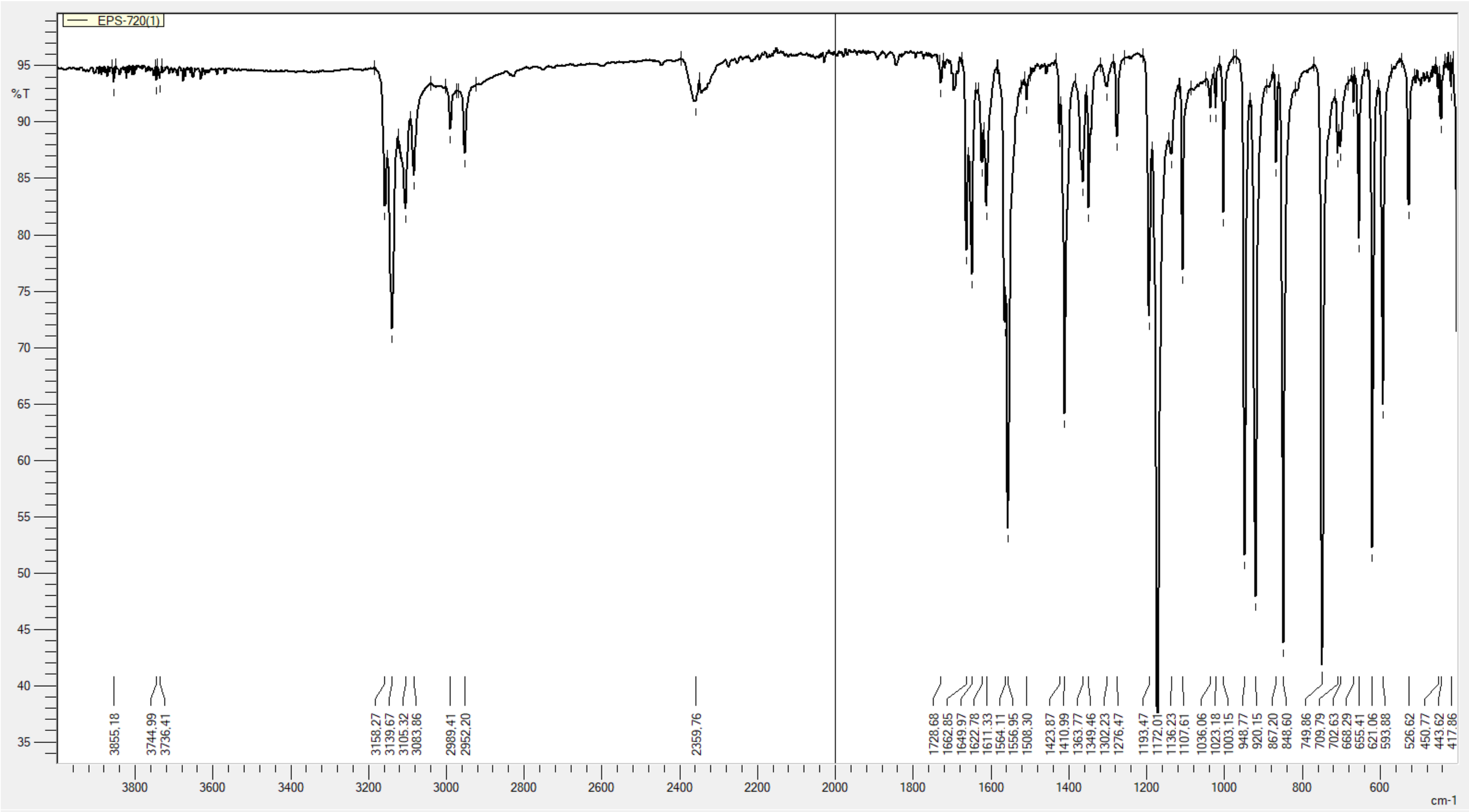


**Figure S17.** IR spectra of 3-(cyanomethyl)-1-vinyl-1*H*-imidazol-3-ium tetrachloridoferrate(III) (**9**).

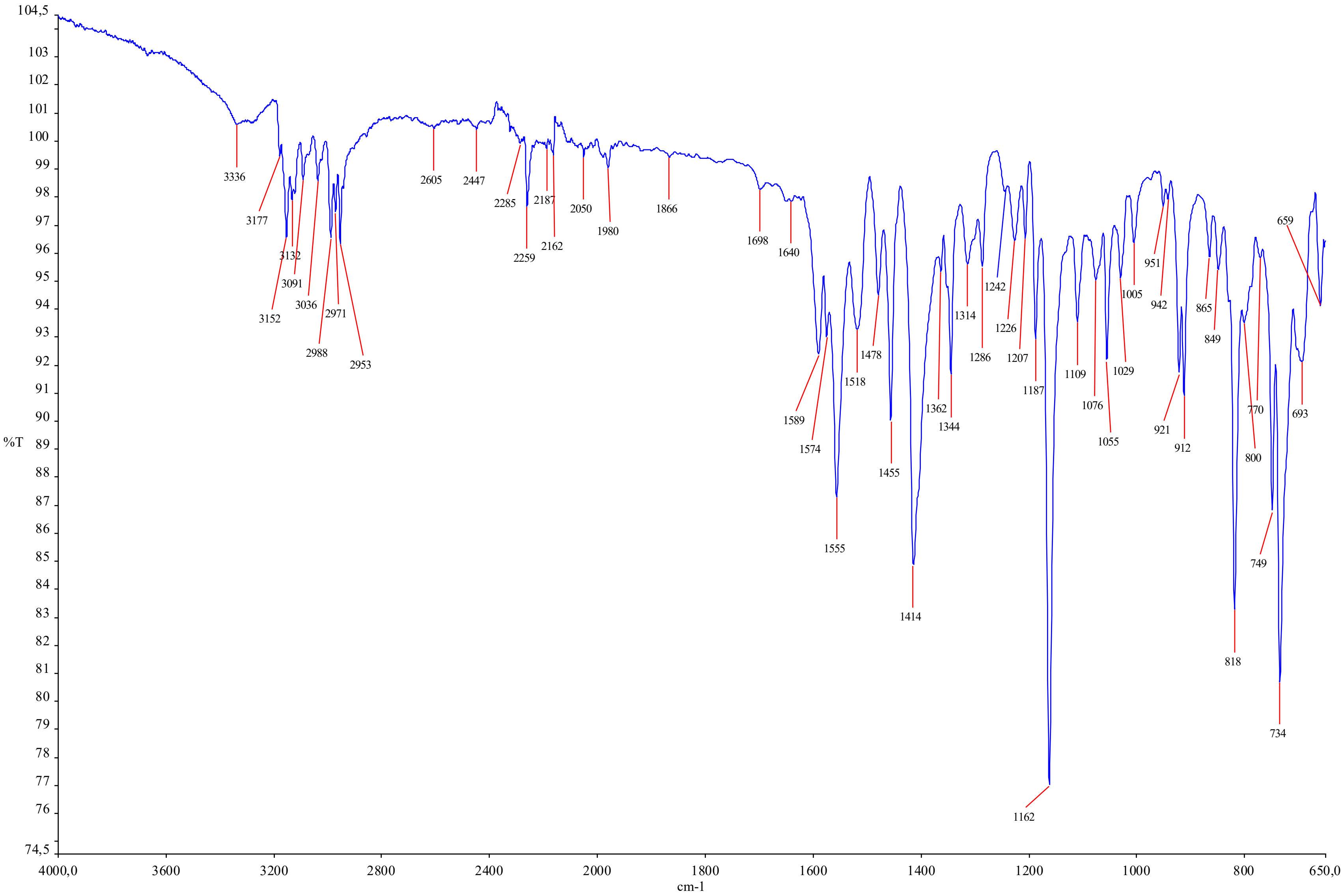


**Figure S18.** FTIR spectra of 1-(2-cyanoethyl)-3-(cyanomethyl)-1*H*-imidazol-3-ium tetrachloridoferrate(III) (**10**).

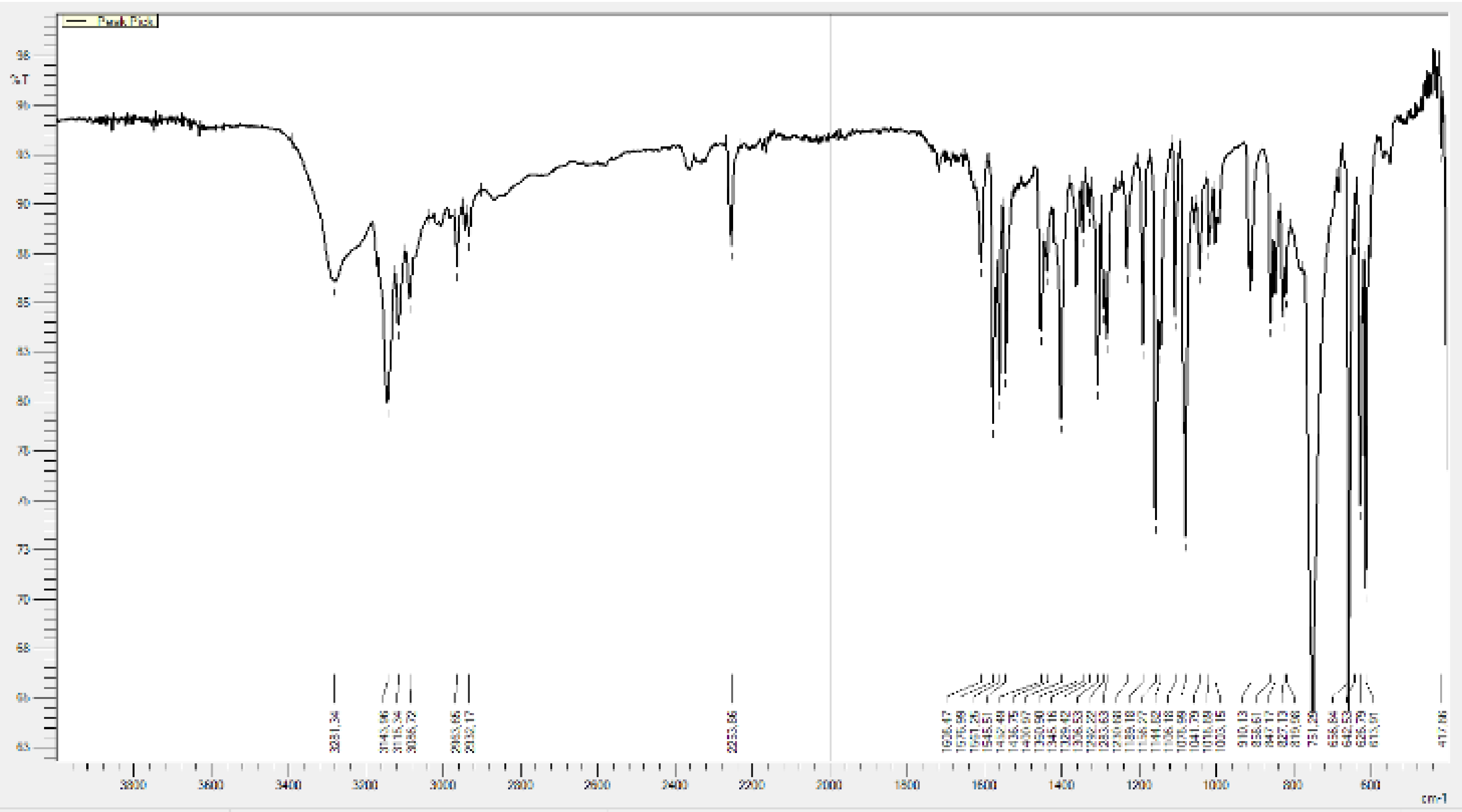


**Figure S19.** IR spectra of 1,3-bis(2-cyanoethyl)-1*H*-imidazol-3-ium tetrachloridoferrate(III) (**11**).

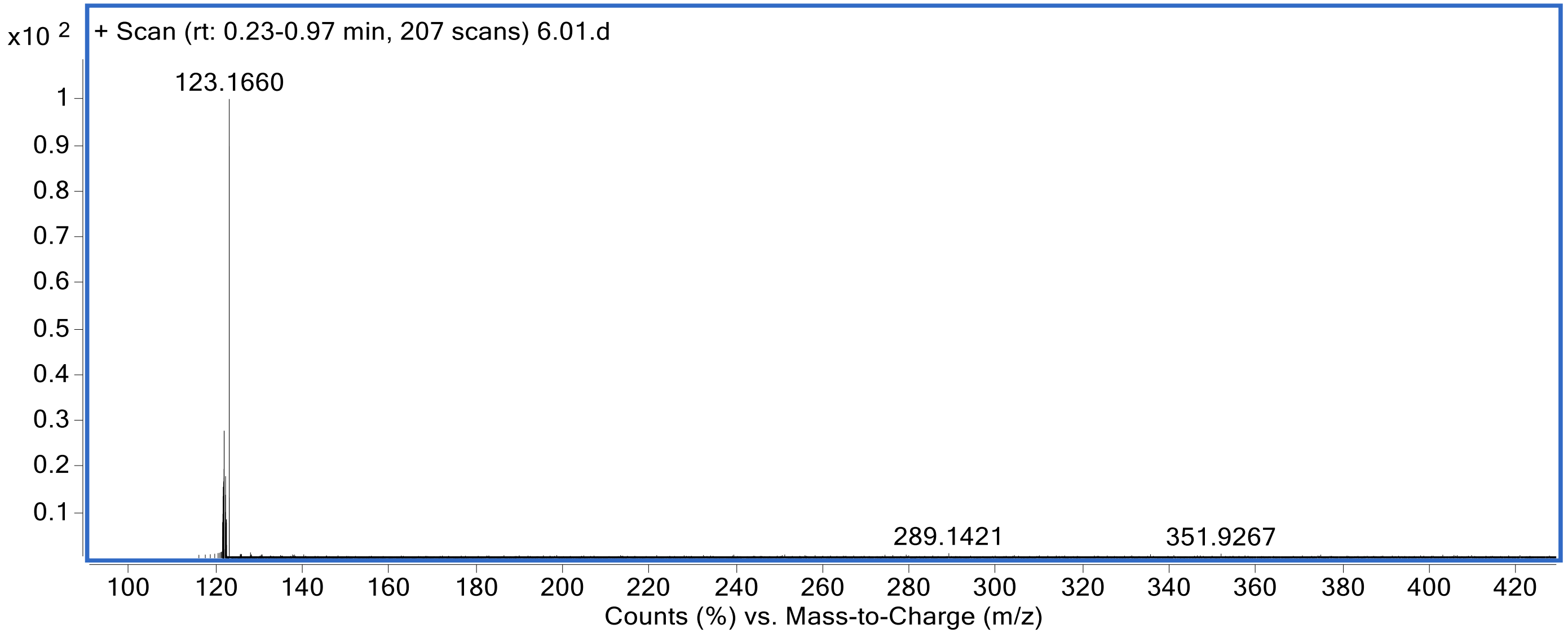


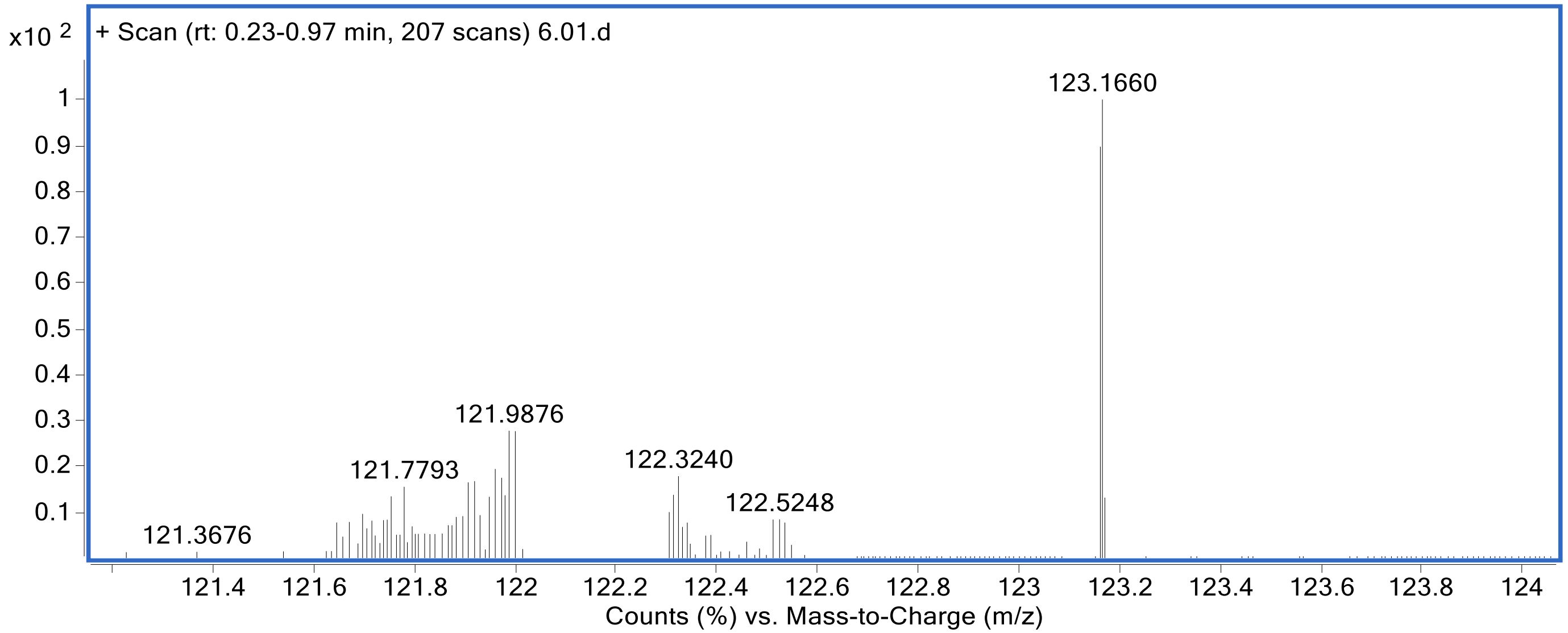


**Figure S20.** ESI+Q/TOF MS of 3-(cyanomethyl)-1-methyl-1*H*-imidazol-3-ium tetrachloridoferrate(III) **8.**

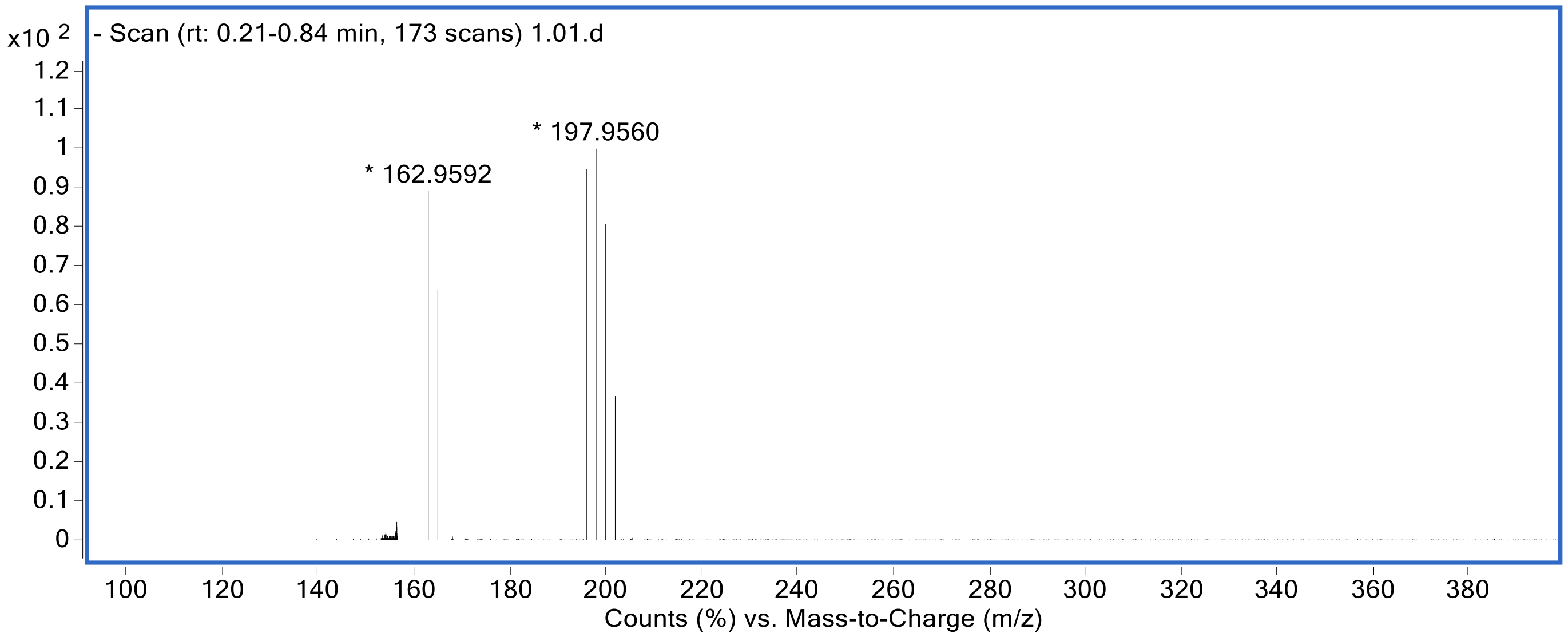


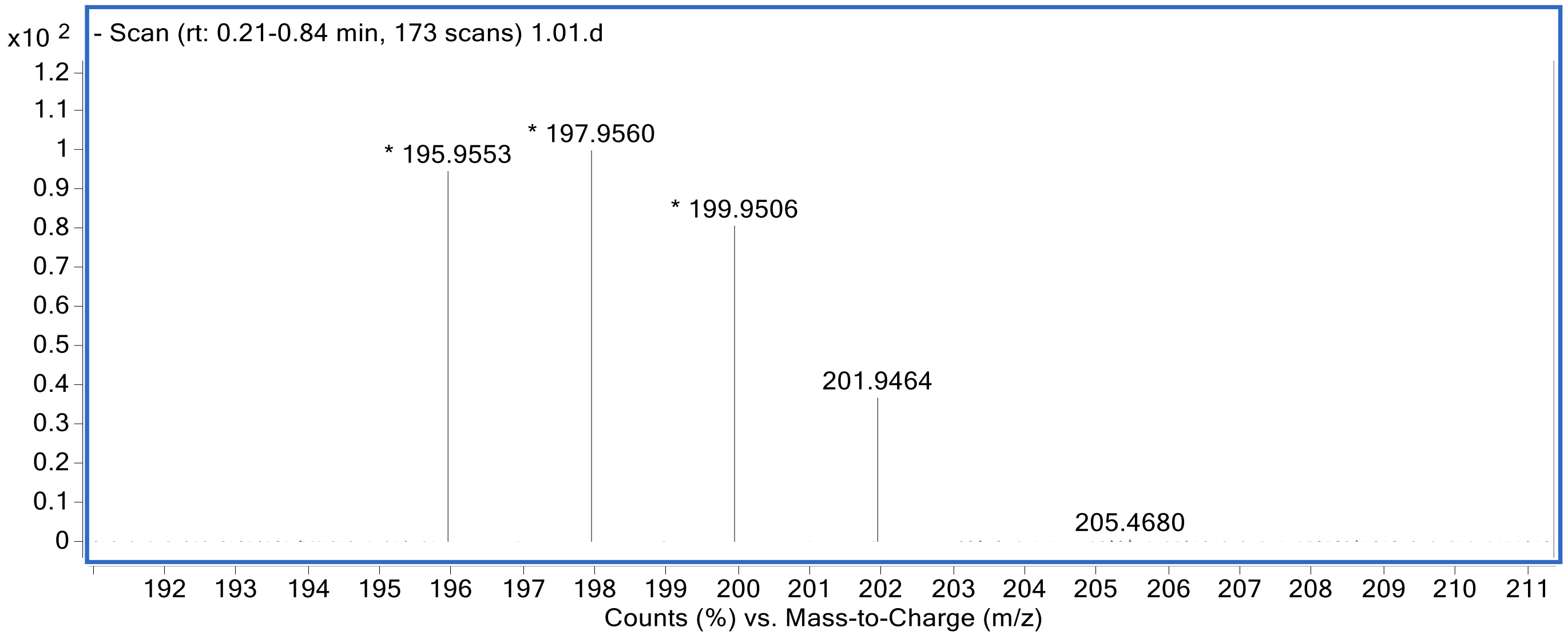


**Figure S21.** ESI-Q/TOF MS of 3-(cyanomethyl)-1-methyl-1*H*-imidazol-3-ium tetrachloridoferrate(III) **8.**

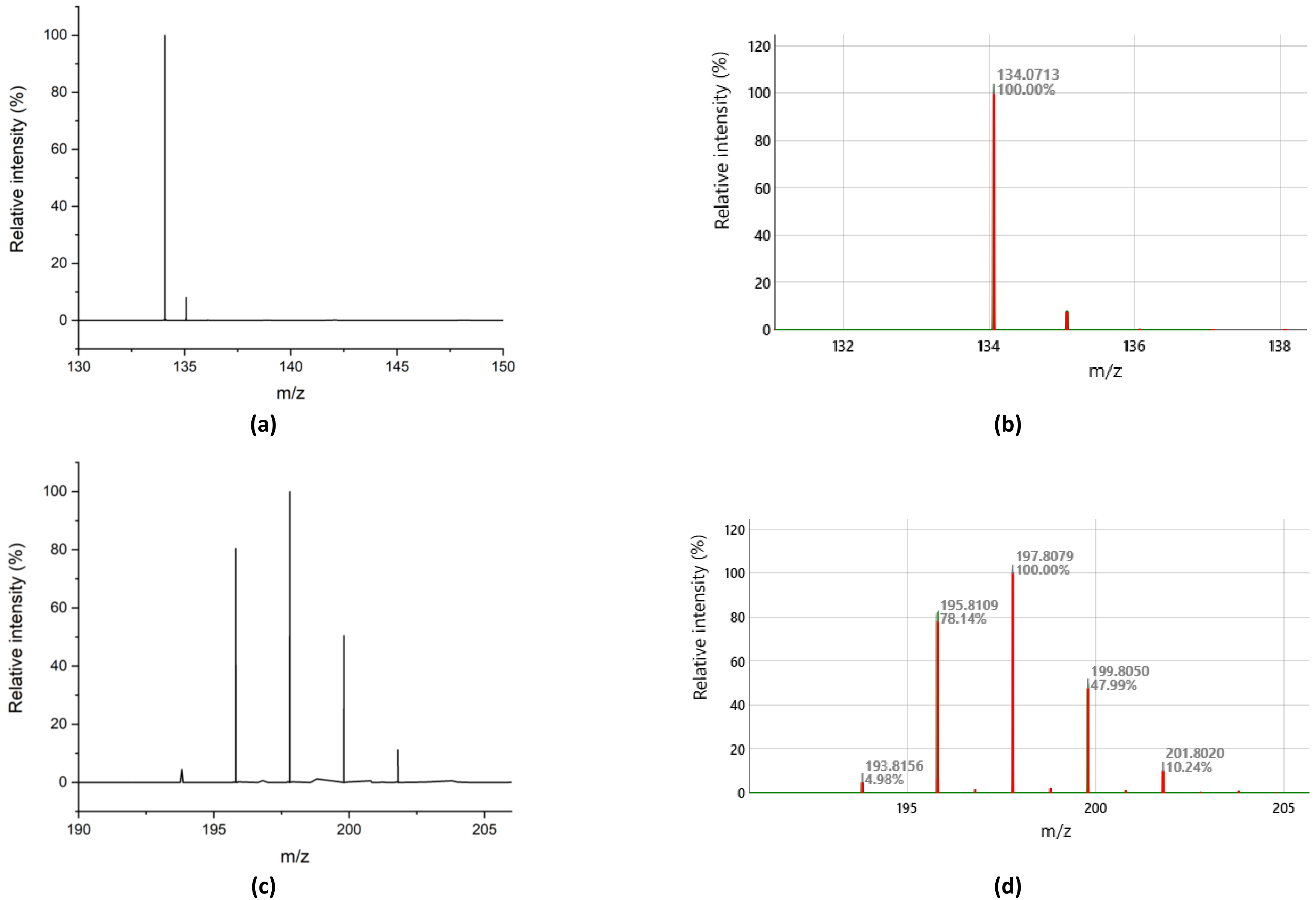


**Figure S22.** Experimental (a, c) and simulated (b,d) ESI-MS isotope patterns for the cationic fragment $[C_7H_8N_3]^+$ in ESI(+) mode (*m/z* 134.07) and the anionic fragment $[FeCl_4]^-$ in ESI(-) mode (*m/z* 197.81) in **9**.

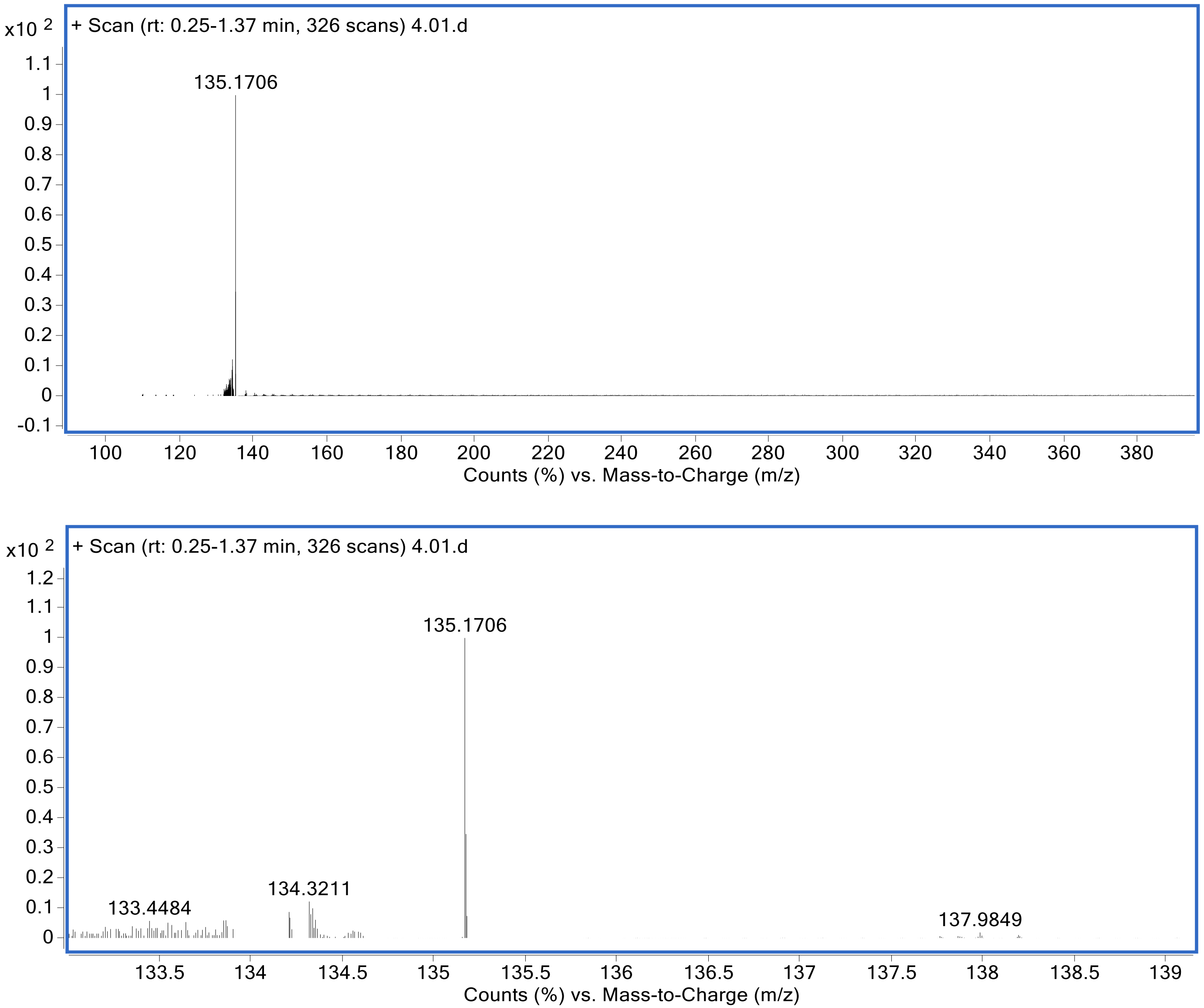


**Figure S23.** ESI+Q/TOF MS of 3-(cyanomethyl)-1-vinyl-1*H*-imidazol-3-ium tetrachloridoferrate(III) **9.**

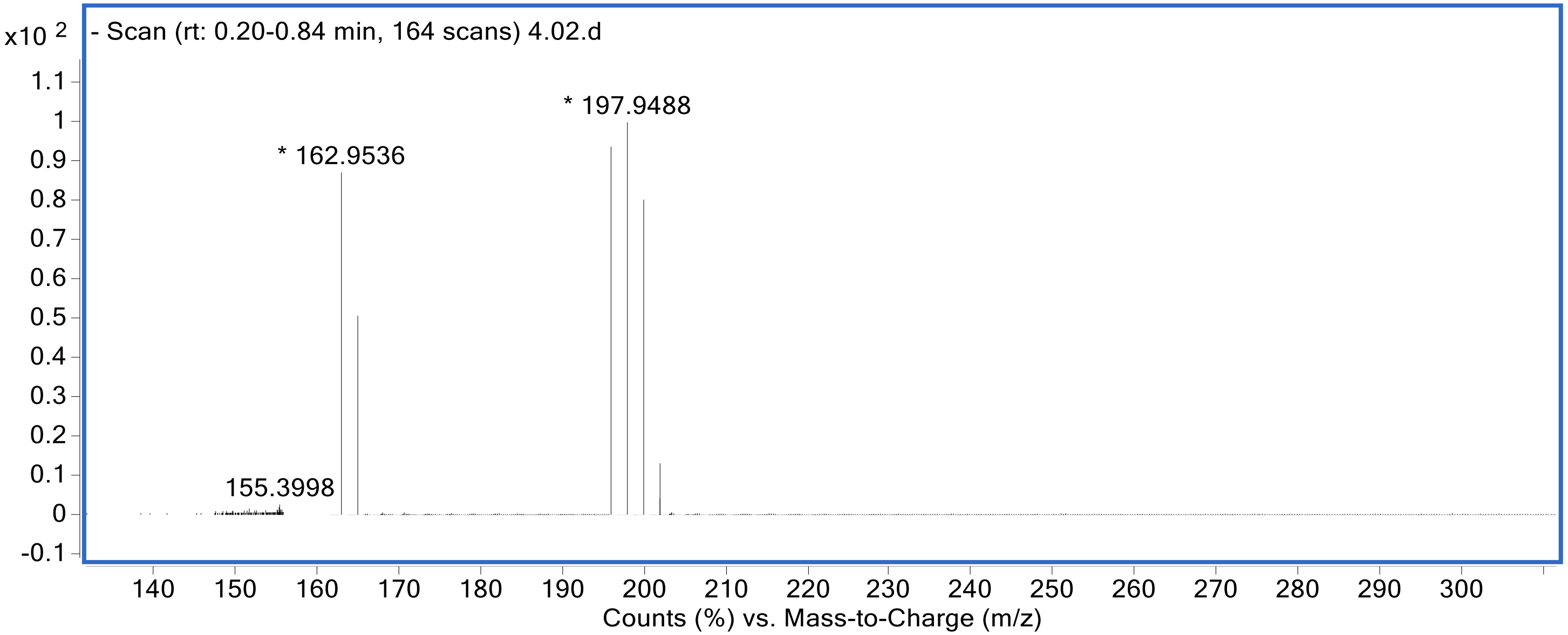


**Figure S24.** ESI-Q/TOF MS of 3-(cyanomethyl)-1-vinyl-1*H*-imidazol-3-ium tetrachloridoferrate(III) **9.**

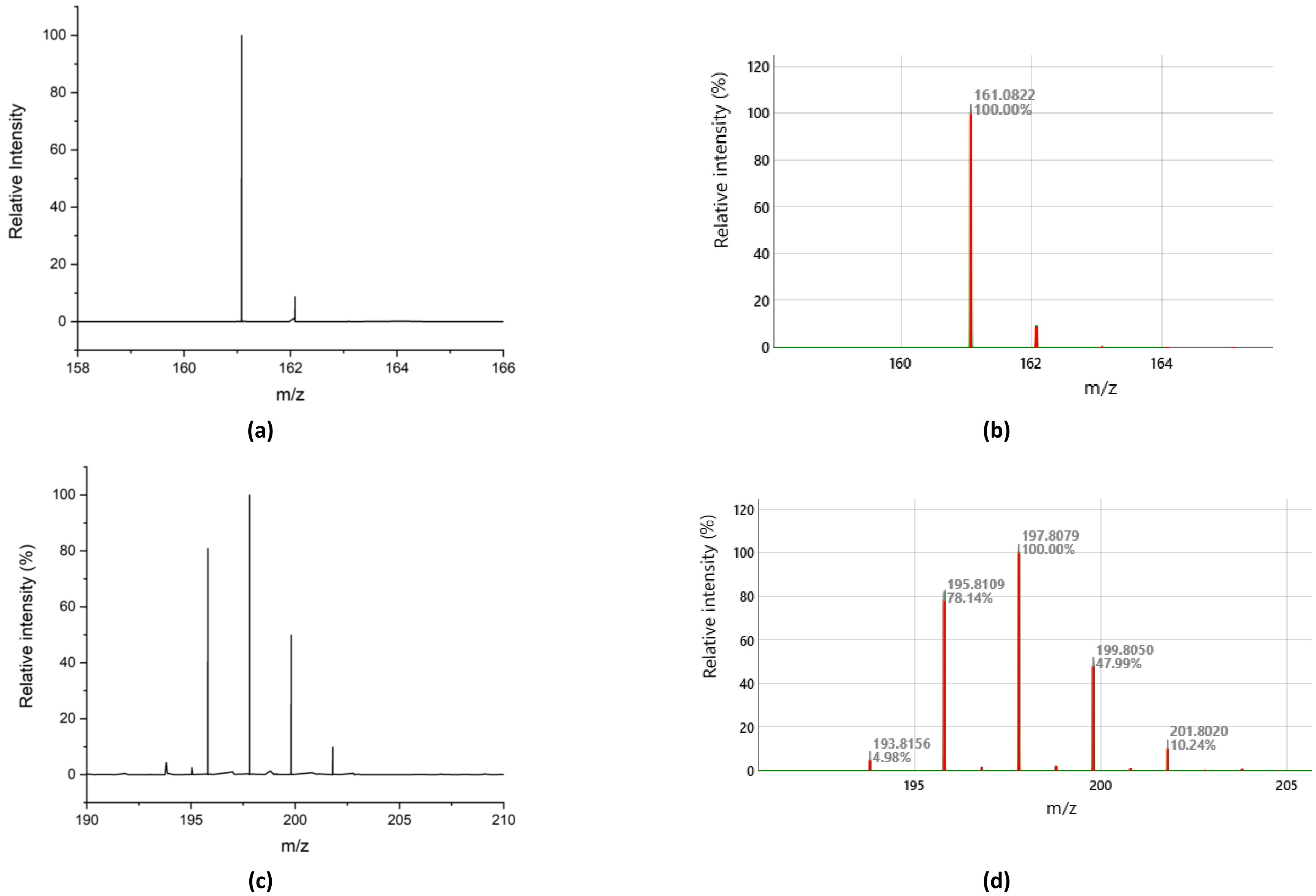


**Figure S25.** Experimental (a, c) and simulated (b,d) ESI-MS isotope patterns for the cationic fragment $[C_8H_9N_4]^+$ in ESI(+) mode (*m/z* 161.08) and the anionic fragment $[FeCl_4]^-$ in ESI(-) mode (*m/z* 197.81) in **10**.

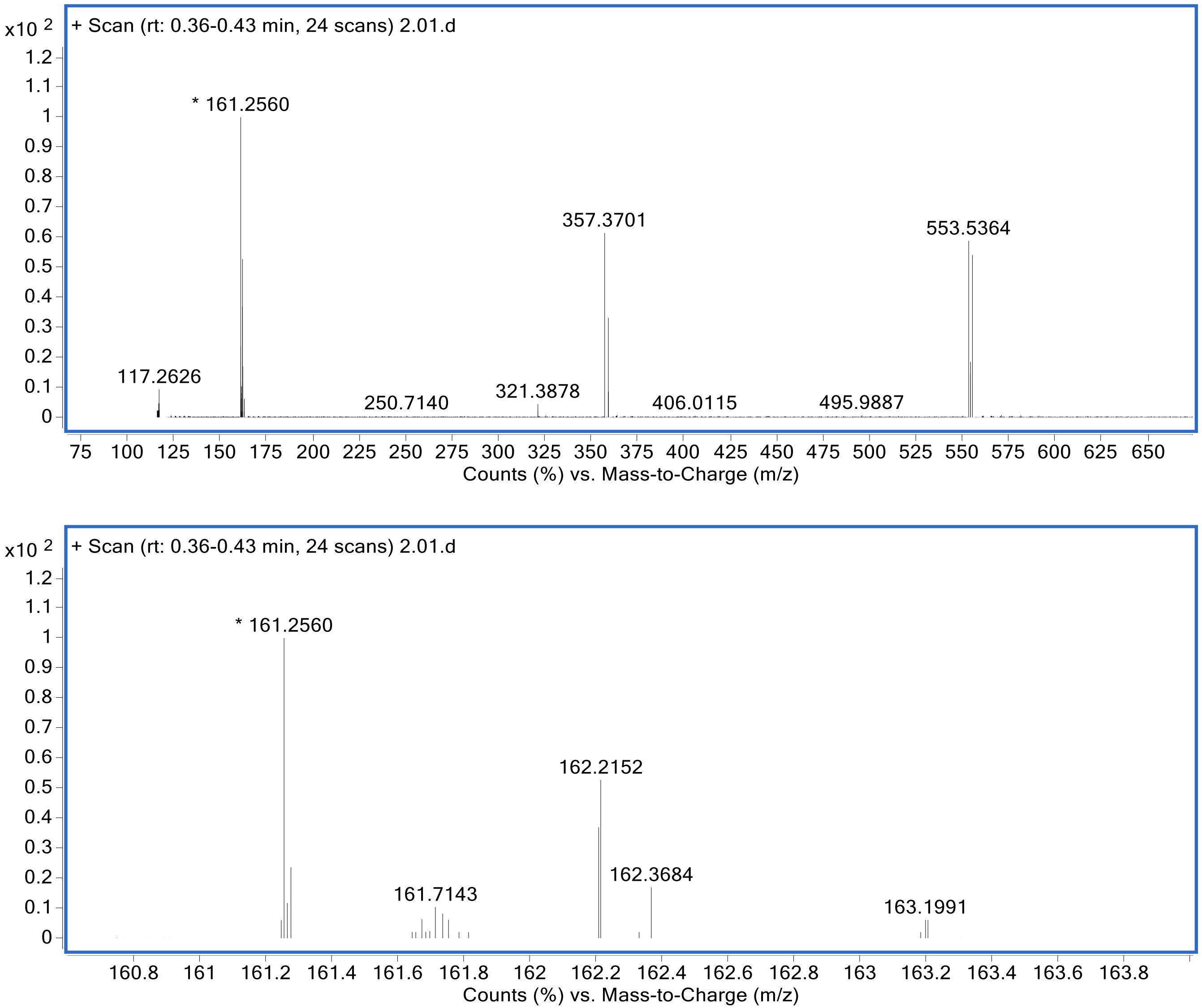


**Figure S26.** ESI+Q/TOF MS of 1-(2-cyanoethyl)-3-(cyanomethyl)-1*H*-imidazol-3-ium tetrachloridoferrate(III) **10**.

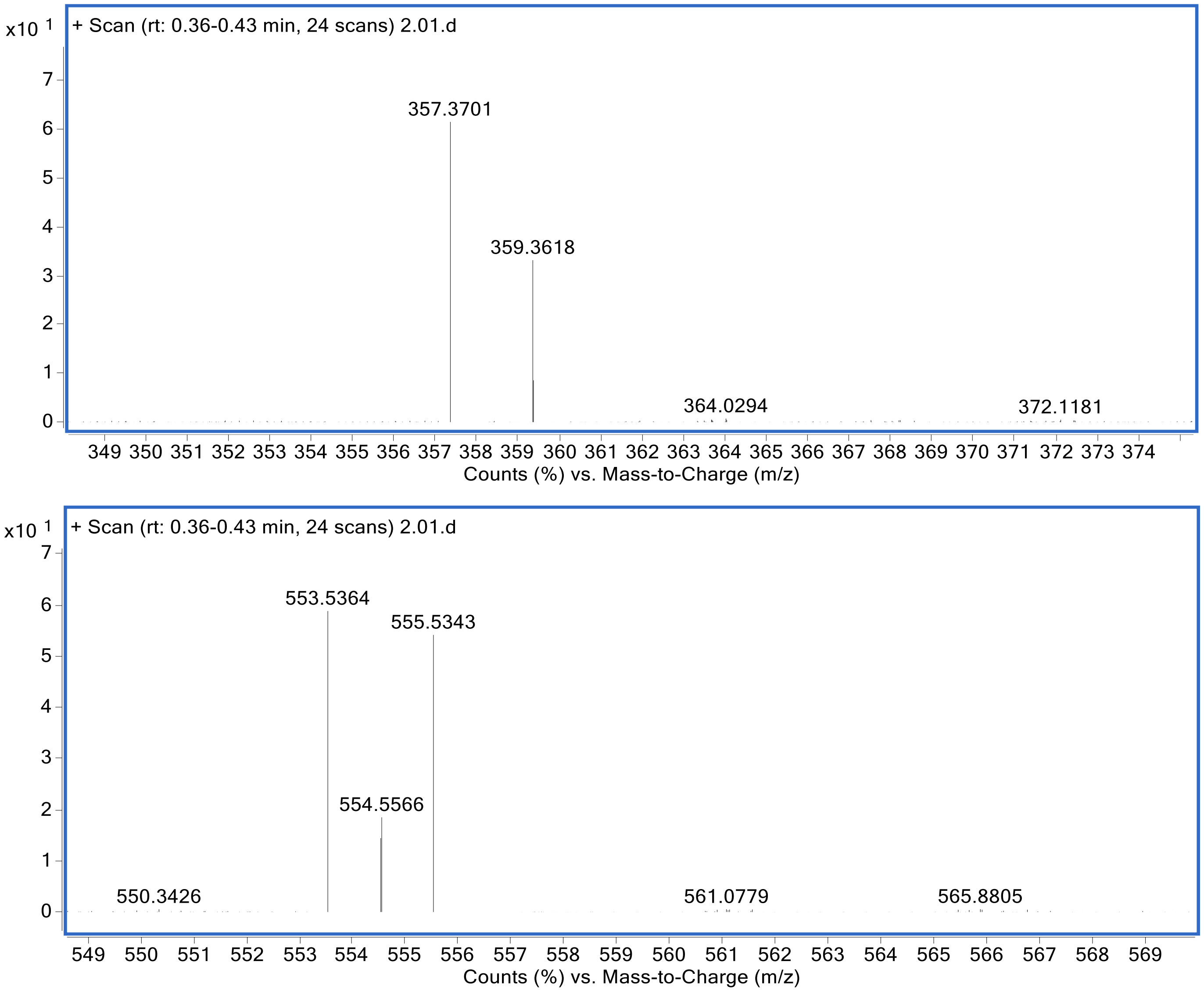


**Figure S27.** ESI+Q/TOF MS of 1-(2-cyanoethyl)-3-(cyanomethyl)-1*H*-imidazol-3-ium tetrachloridoferrate(III) **10**.

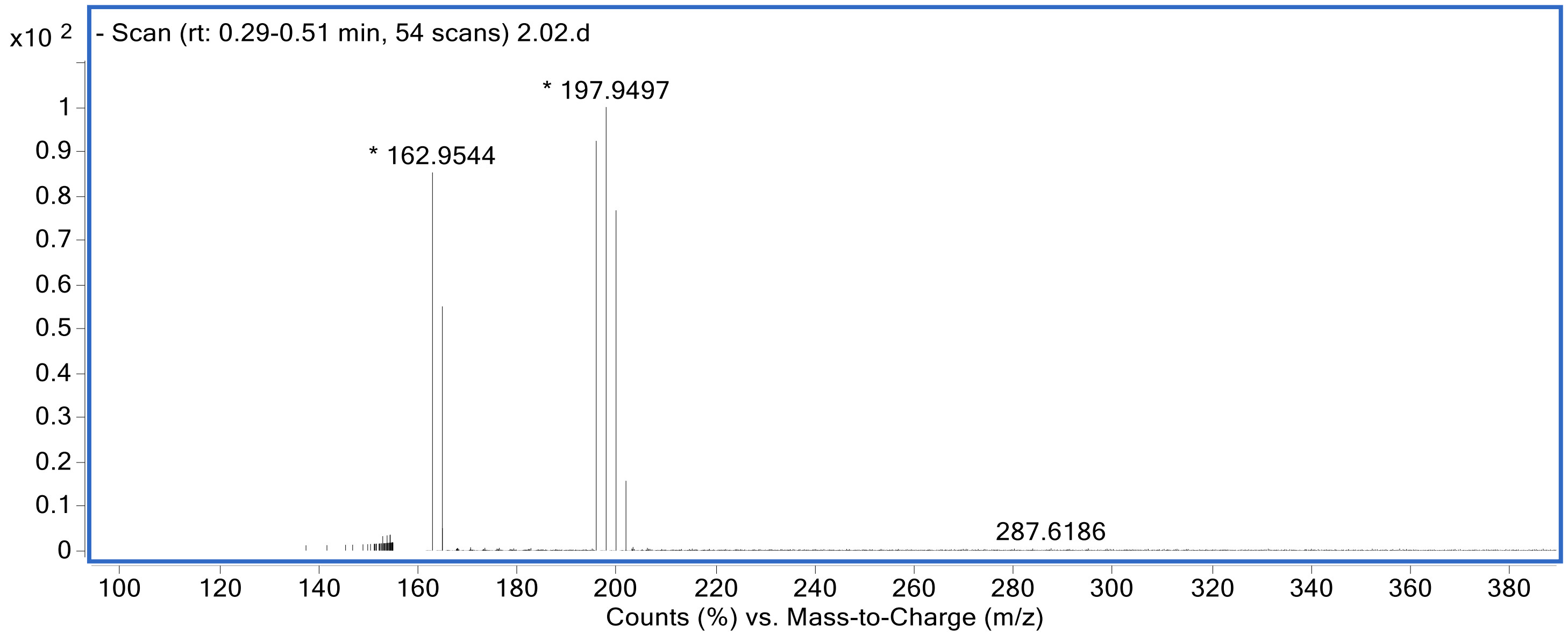


**Figure S28.** ESI-Q/TOF MS of 1-(2-cyanoethyl)-3-(cyanomethyl)-1*H*-imidazol-3-ium tetrachloridoferrate(III) **10**.

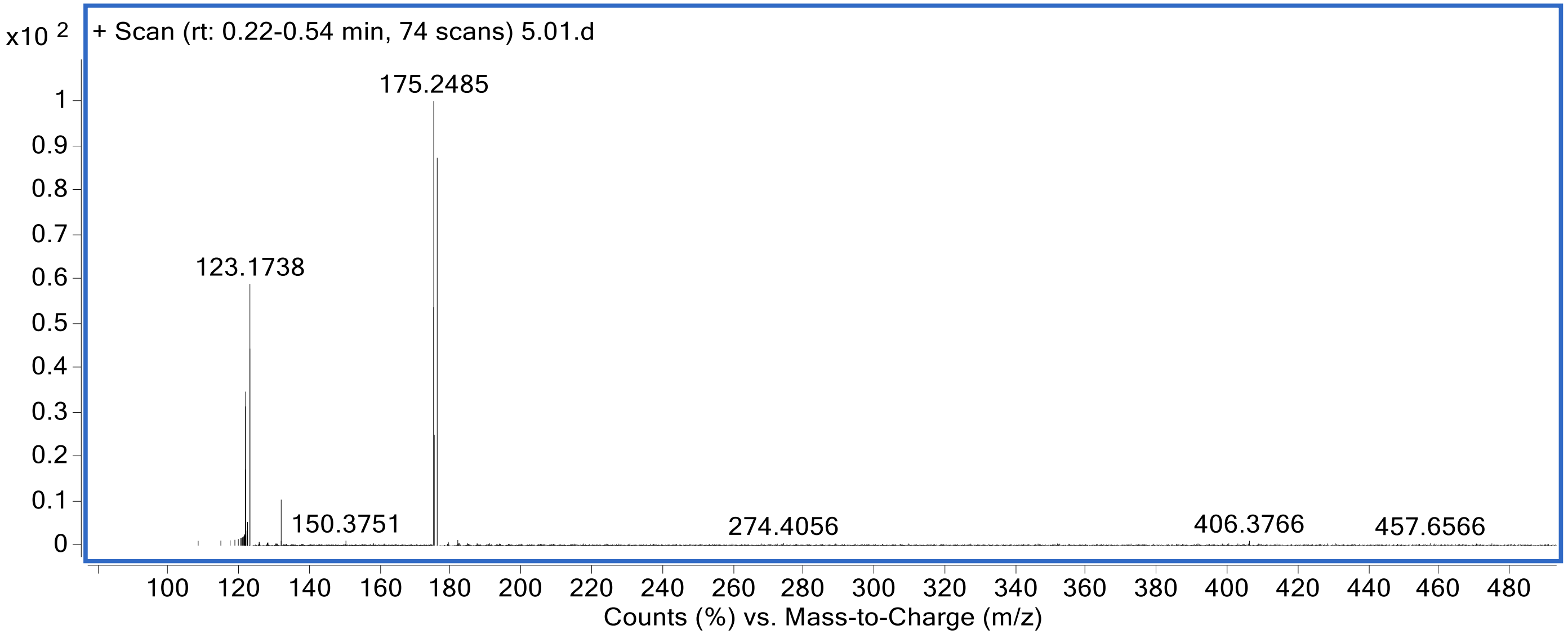


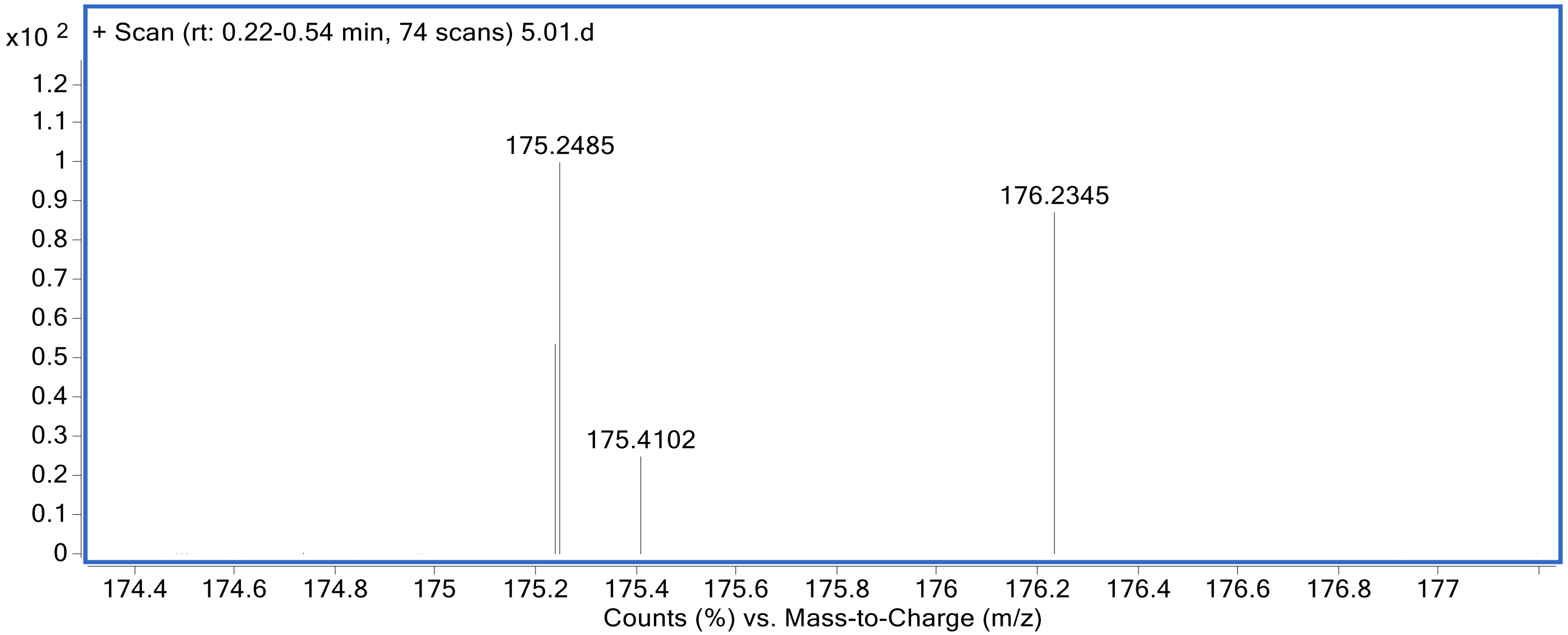


**Figure S29.** ESI+Q/TOF MS of 1,3-bis(2-cyanoethyl)-1*H*-imidazol-3-ium tetrachloridoferrate(III) **11**.
